# Data-Driven Insights into the Transition-Metal-Catalyzed Asymmetric Hydrogenation of Olefins

**DOI:** 10.1021/acs.joc.4c01396

**Published:** 2024-08-16

**Authors:** Sukriti Singh, José Miguel Hernández-Lobato

**Affiliations:** Department of Engineering, University of Cambridge, Cambridge CB2 1PZ, U.K.

## Abstract

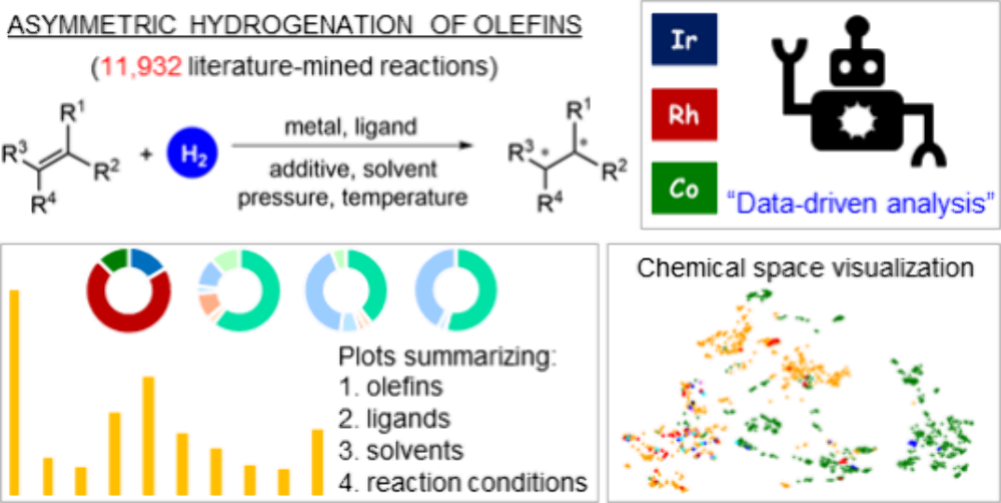

The transition-metal-catalyzed asymmetric hydrogenation of olefins is one of the key transformations with great utility in various industrial applications. The field has been dominated by the use of noble metal catalysts, such as iridium and rhodium. The reactions with the earth-abundant cobalt metal have increased only in recent years. In this work, we analyze the large amount of literature data available on iridium- and rhodium-catalyzed asymmetric hydrogenation. The limited data on reactions using Co catalysts are then examined in the context of Ir and Rh to obtain a better understanding of the reactivity pattern. A detailed data-driven study of the types of olefins, ligands, and reaction conditions such as solvent, temperature, and pressure is carried out. Our analysis provides an understanding of the literature trends and demonstrates that only a few olefin–ligand combinations or reaction conditions are frequently used. The knowledge of this bias in the literature data toward a certain group of substrates or reaction conditions can be useful for practitioners to design new reaction data sets that are suitable to obtain meaningful predictions from machine-learning models.

## Introduction

The asymmetric hydrogenation of olefins (AHO) is one of the most fundamental transformations for the synthesis of chiral molecules.^1,2^ The popularity of this method can be attributed to its atom economy, mild reaction conditions, high enantioselectivity, sustainable strategy, and broad substrate scope.^3^ All of these features together define AHO as an effective tool in stereoselective synthesis, with significance in both academic research and industrial applications.^4,5^ This field has undergone several advancements since early reports on the successful use of chiral bisphosphine ligands in Rh- and Ru-catalyzed asymmetric hydrogenation.^6^ This has led to the development of newer catalysts and chiral ligands, thus greatly increasing the reaction scope by the hydrogenation of diverse olefins in a highly enantioselective manner.^7−9^ While AHO has witnessed rapid progress in the last few decades, some complexities remain. For instance, a majority of the AHO reactions utilize noble transition metal catalysts based on iridium, rhodium, or ruthenium.^10−12^ Also, the asymmetric hydrogenation of unfunctionalized and/or tetrasubstituted olefins has undergone slower growth when compared to di- and trisubstituted olefins.^13^ Thus, the use of earth-abundant metals has become an attractive option with a continuing interest to design catalysts for more challenging olefin substrates.^14,15^

Over the years, numerous catalytic systems have been introduced, with ligands playing a crucial role in achieving chiral induction. The design of chiral metal–ligand complexes to obtain enhanced activity and selectivity has always remained a topic of great interest.^16−18^ In addition, identifying the optimal conditions for a particular reaction, which generally involves the choice of solvents, additives, temperature, pressure, etc., is equally important. The traditional approach to reaction optimization is often time-consuming, and several data-driven strategies have been reported to accelerate the exploration of high-dimensional chemical space.^19−21^ For instance, various examples are available for the applications of machine-learning (ML) methods for catalyst design, reaction optimization, yield, and/or selectivity prediction.^22−26^

The ability of a trained machine-learning model to provide reasonable predictions depends on the data, to a great extent. The lack of diversity and bias in literature-mined data sets toward a certain set of substrates or reaction conditions presents a challenge for data-driven modeling.^27−29^ It has been shown that the ML models trained on such data sets may simply capture literature trends and provide no better insights than the models suggesting the most popular reaction conditions.^30^ Thus, the need to routinely report low-output reactions and use experimental designs that cover a broader chemical space are identified as crucial factors for more reliable predictive models.^31−35^ Therefore, it is extremely important to understand the inner workings of the data to enable the development of more useful machine-learning and other data-driven modeling approaches for synthetic chemistry.^36−38^

The wide applicability of AHO has led to the availability of large amounts of literature data. This provides a unique opportunity to apply data-rich approaches for reaction development.^39,40^ In this regard, we performed a detailed data-driven analysis of AHO reactions catalyzed by iridium, rhodium, and cobalt metal catalysts. These transition metals are selected for analysis as they belong to the same group in the periodic table and might often share similar reactivity patterns. Therefore, the insights from the significant advances in Ir- and Rh-catalyzed AHO reactions could be emulated to develop cost-effective Co catalysts. Herein, the trends discovered by analyzing the data are discussed in terms of metal catalysts, types of olefins, ligands, and reaction conditions. We believe that this study will expand the understanding of the interplay between various reaction parameters along with providing directions for further studies. Furthermore, it could be of great utility for building ML models suited to literature data sets along with better evaluation metrics.

## Results and Discussion

**Figure 1:**
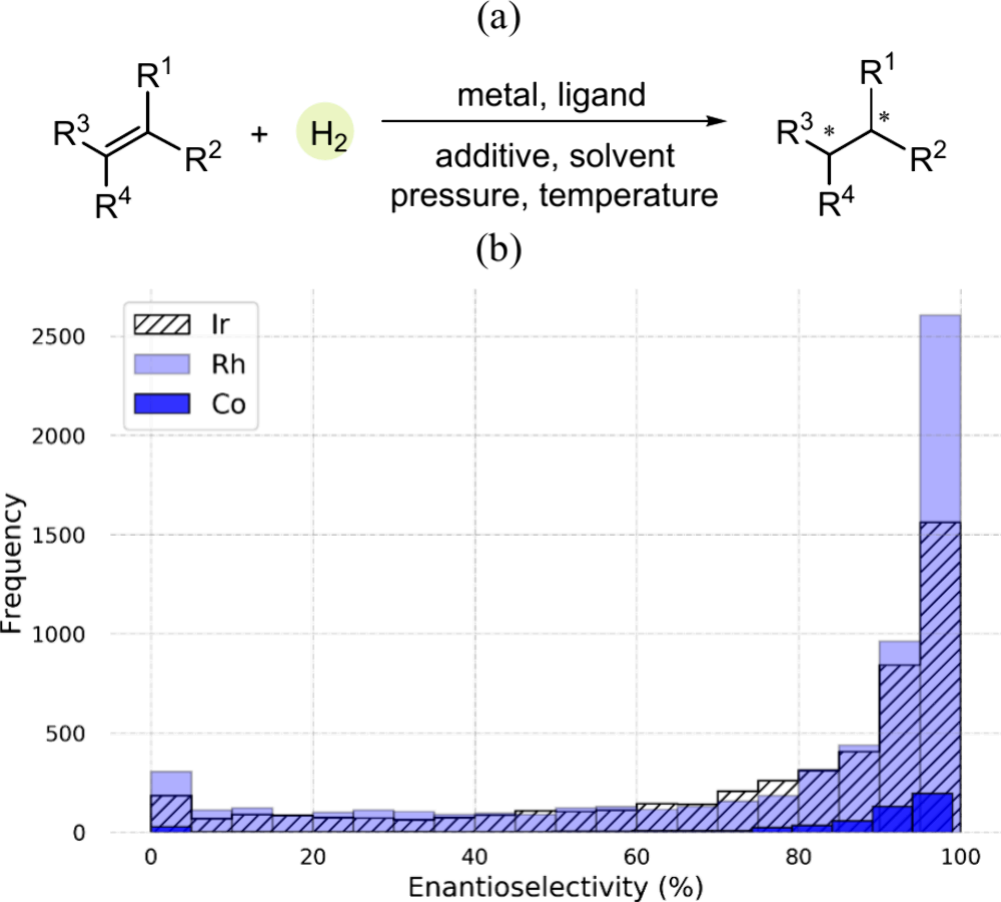
(a) A general reaction scheme for the AHO. (b) Distribution of the reactions across various enantioselectivity regimes.

This analysis is carried out using a recently published literature-mined reaction data set focusing on transition-metal-catalyzed AHO (Figure 1a).^41,42^ The full data set consists of 12,619 reactions with 2754 olefin substrates and 1686 ligands. For each reaction, the simplified molecular-input line-entry system (SMILES)^43^ of substrates, ligands, solvents, additives, and products is provided along with the following experimental variables: pressure, temperature, and catalyst loading. The reaction performance is reported in terms of enantiomeric excess (ee) and yield and conversion (if available). Regarding the distribution of transition metals, rhodium and iridium are the most widely used catalysts, constituting 53.1 and 37.9%, respectively. However, cobalt constitutes a mere 1% of all of the reported catalysts. As the data set has only a few examples of cobalt-catalyzed AHO reactions, we manually extracted more data from studies published during the last couple of years.

The AHO has three main reaction components: an olefin substrate, a metal, and a ligand. In this study, our primary focus is on the AHO catalyzed by iridium, rhodium, and cobalt metal catalysts. The data set contains 5009 Ir-catalyzed AHO reactions with 1181 olefin substrates and 805 ligands. For Rh, there are 6391 reactions with 1386 distinct olefin substrates and 721 ligands. Similarly, there are 532 reactions with Co catalysts comprising 366 olefins and 58 ligands. The reaction performance data in terms of enantioselectivity shows a similar trend for all three metal catalysts and is highly skewed toward examples with a high %ee (Figure 1b). This bias in reporting the highest achievable yields and/or selectivities is a common concern and impacts the accuracy of data-driven or machine-learning approaches.^44,45^ For improved clarity, we have categorized the discussion into four key sections, as described below.

### Olefins

**Figure 2:**
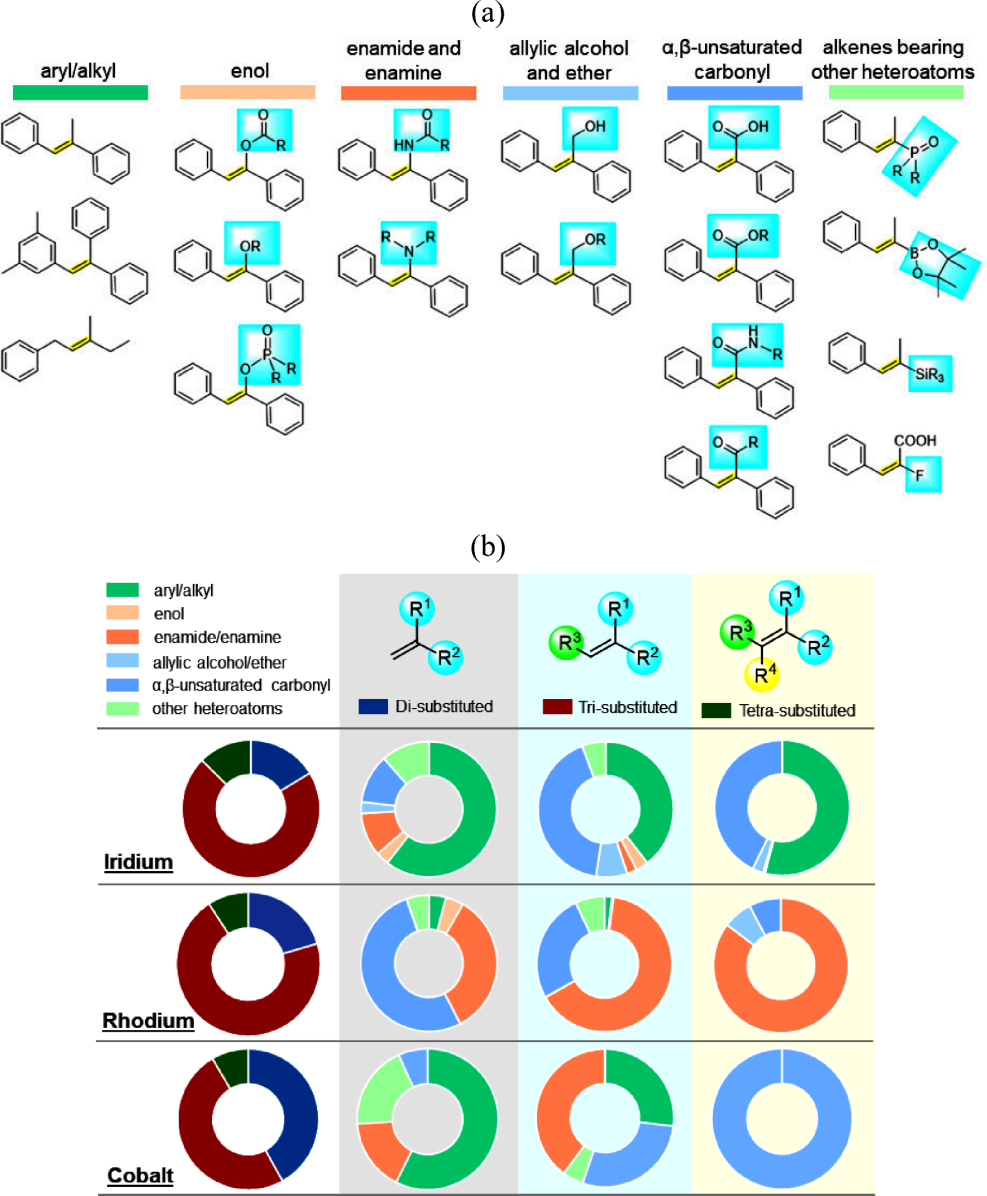
(a) Representative examples of the types of alkene substrates used in the transition-metal-catalyzed asymmetric hydrogenation reaction. (b) Distribution of the types of alkene substrates used with Ir, Rh, and Co metal catalysts in the AHO.

To begin with, we classify olefin substrates broadly into three categories, i.e., disubstituted, trisubstituted, and tetrasubstituted alkenes. It can be noted from Figure 2b that trisubstituted olefins are the dominant substrate type, with over 60% reactions for both Ir and Rh, followed by disubstituted olefins. However, in the case of Co, both di- and trisubstituted olefins are distributed almost equally. The difficulty in the asymmetric hydrogenation of tetrasubstituted olefins is evident from the least number of reactions with all three metal catalysts.

A more fine-grained analysis is carried out by further dividing each of the three olefin types into six classes. These are aryl- and alkyl-substituted alkenes, enols, enamides and enamines, allylic alcohols and ethers, α,β-unsaturated carbonyls, and alkenes bearing other heteroatoms (Figure 2a). The aryl- and alkyl-substituted alkenes consist of aryl–alkyl, aryl–aryl, and alkyl–alkyl alkenes. They form the benchmark substrates in estimating the competence of new catalytic systems in hydrogenating minimally functionalized olefins. The asymmetric hydrogenation of enols has been used as an alternative to the asymmetric hydrogenation of ketones. Some of the popular enol-type substrates include enol esters, enol carbamates, enol phosphinates, enol phosphonates, enol ethers, and silyl enol ethers. The asymmetric hydrogenation of enamides and enamines is an alternative approach to the asymmetric hydrogenation of imines. Allyl alcohols and ethers are another class of substrates used in AHO. α,β-Unsaturated carbonyls have been used very frequently in AHO; some examples include α,β-unsaturated carboxylic acids, esters, amides, and ketones (Figure 2a). Stereogenic centers bearing heteroatoms such as phosphorus, boron, fluorine, silicon, etc., have also been utilized in asymmetric hydrogenation.

The Ir-catalyzed asymmetric hydrogenation of disubstituted olefins is dominated by aryl- and alkyl-substituted alkenes, which constitute 60% of the reported reactions. The remaining 40% reactions comprise the other five olefin types, with enols and allylic alcohols having the least number of examples. On comparison with Rh, a different trend is noted. α,β-Unsaturated carbonyls are the most used substrate type with over 50% reactions, followed by enamides and enamines. In contrast to Ir, only 4% of the reactions use aryl- and alkyl-substituted alkenes with a Rh catalyst.

For trisubstituted olefins, aryl- and alkyl-substituted alkenes and α,β-unsaturated carbonyls are the primary substrate types used with Ir. On the other hand, the hydrogenation of enamides and enamines is the most frequent reaction with the Rh catalyst, followed by a significant number of examples with α,β-unsaturated carbonyls (Figure 2b). Similar to di- and trisubstituted olefins, aryl- and alkyl-substituted alkenes are majorly used with Ir in reactions with tetrasubstituted olefins. The distribution of α,β-unsaturated carbonyls remains identical to that of trisubstituted olefins. In the case of Rh, enamides are the dominant substrate type with tetrasubstituted olefins. It is quite evident that the type of tetrasubstituted olefin substrates that can be effectively hydrogenated is still limited. We also note that many of the reported olefins have both enamides and α,β-unsaturated carbonyls together. Although enols have only a few reactions with both Ir and Rh, enol ethers in combination with α,β-unsaturated carbonyls are common. In addition, alkenes bearing fluorine atoms along with α,β-unsaturated carbonyls are found to be frequent in tetrasubstituted olefins.^46^

The cobalt-catalyzed asymmetric hydrogenation of disubstituted olefins is dominated by aryl- and alkyl-substituted alkenes, following the same trend as that for Ir catalysts (Figure 2b). For trisubstituted olefins, the types of substrates used with Co catalysts appear to be a combination of both Ir and Rh with aryl- and alkyl-substituted alkenes, enamides, and α,β-unsaturated carbonyls. In the case of tetrasubstituted olefins, the reactions are reported almost exclusively with α,β-unsaturated carbonyls, especially α,β-unsaturated carboxylic acids. Of note, because Co-catalyzed AHO reactions have limited examples, the analysis presented should be seen as an early advancement in this field. The top five olefins with the maximum number of Ir-, Rh-, and Co-catalyzed AHO reactions are shown in the Supporting Information (Figure S1). A comparison of the median enantioselectivity of each olefin type for all three metal catalysts is presented in Figure S2.

**Figure 3:**
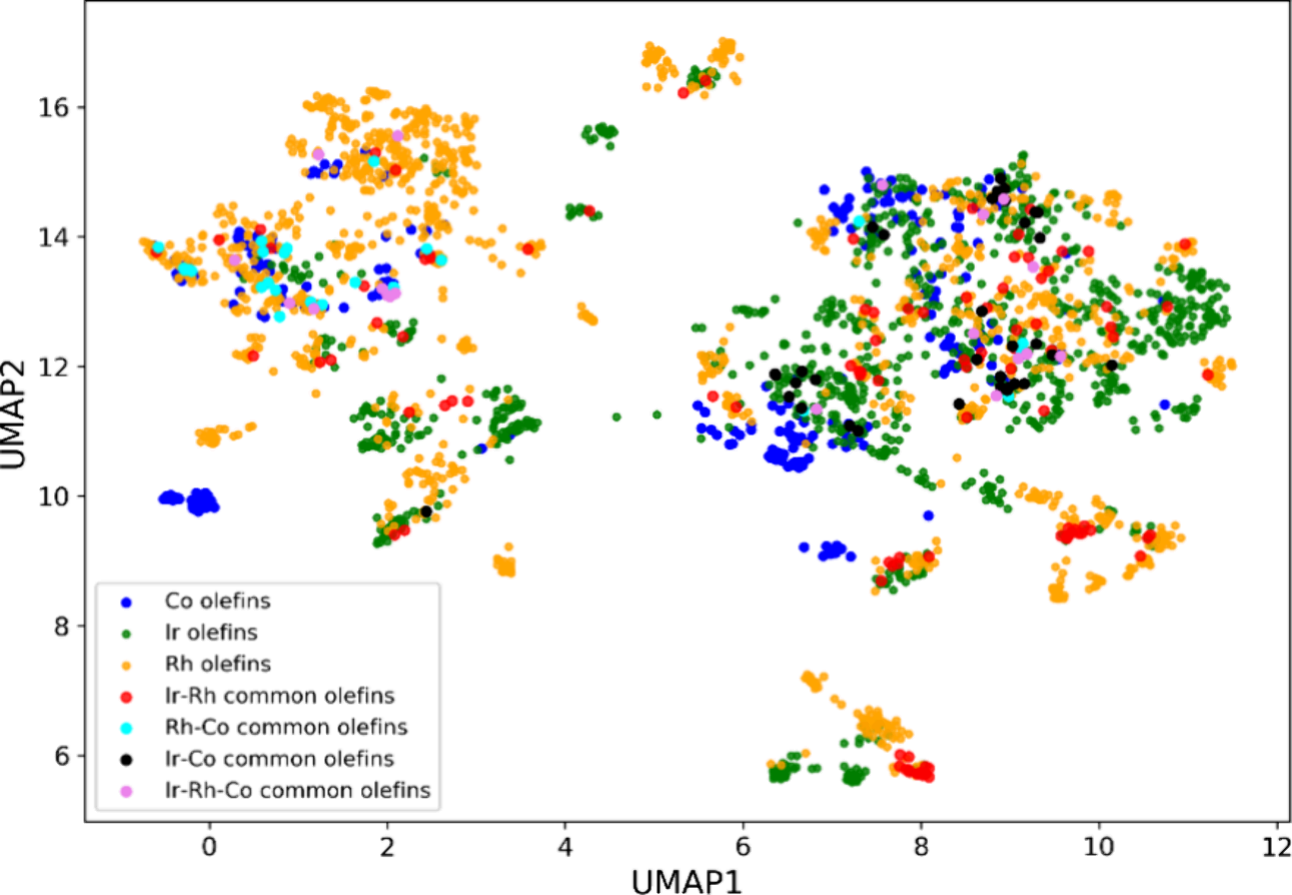
UMAP plot of the chemical space of olefins used in Ir-, Rh-, and Co-catalyzed asymmetric hydrogenation. The *x*- and *y*-axes correspond to the two UMAP components obtained after dimensionality reduction.

Next, we visualized the chemical space covered by the olefins utilized in asymmetric hydrogenation catalyzed by all three metal catalysts. For this purpose, we used the uniform manifold approximation and projection (UMAP) plot, which is a nonlinear dimensionality reduction technique for visualizing complex high-dimensional data in lower dimensions.^47^ We converted the SMILES string of the olefins into 166-bit 2D structural fingerprints, known as molecular access system keys,^48^ which serve as inputs to the UMAP plot. Figure 3 shows the UMAP plot for the alkene substrates used with Ir (shown in green), Rh (shown in orange), Co (shown in blue), and the olefins common among these metal catalysts (see Figure S2 for individual UMAP plots). First, the olefins common to Ir–Rh, Ir–Co, and Rh–Co are scarce (Table S1). Although there is a significant overlap in the chemical space covered by Ir and Rh olefins, the enamides used in reactions with Rh form a distinct cluster (top left region in Figure 3). Also, the olefins used with Co catalysts are not spread uniformly across the chemical space and provide an opportunity for further exploration. We also visualized the chemical space covered by the olefins based on their average enantioselectivity values (Figure S4). Similar to the distribution of reaction data in terms of enantioselectivity (Figure 1b), the olefins are also noted to be skewed toward higher enantioselectivity values.

In summary, several important insights into the type of olefin substrates suitable for Ir-, Rh-, and Co-catalyzed asymmetric hydrogenation are gathered from the data-driven analysis. The Rh-catalyzed asymmetric hydrogenation generally requires a coordinating functional group, such as carboxylic acid and amide, in the vicinity of the double bond. Only a few reactions are present for the asymmetric hydrogenation of minimally functionalized olefins using Rh catalysts. In contrast, asymmetric hydrogenation with Ir catalysts is primarily with unfunctionalized and/or minimally functionalized olefins, for example, aryl- and alkyl-substituted alkenes. Additionally, there are sufficient examples of asymmetric hydrogenation of functionalized olefins, such as α,β-unsaturated carbonyls, using Ir catalysts. Interestingly, the olefins used in Co-catalyzed asymmetric hydrogenation have characteristics common to both Ir and Rh metal catalysts and, therefore, can be seen as viable alternatives.

### Ligands

**Figure 4:**
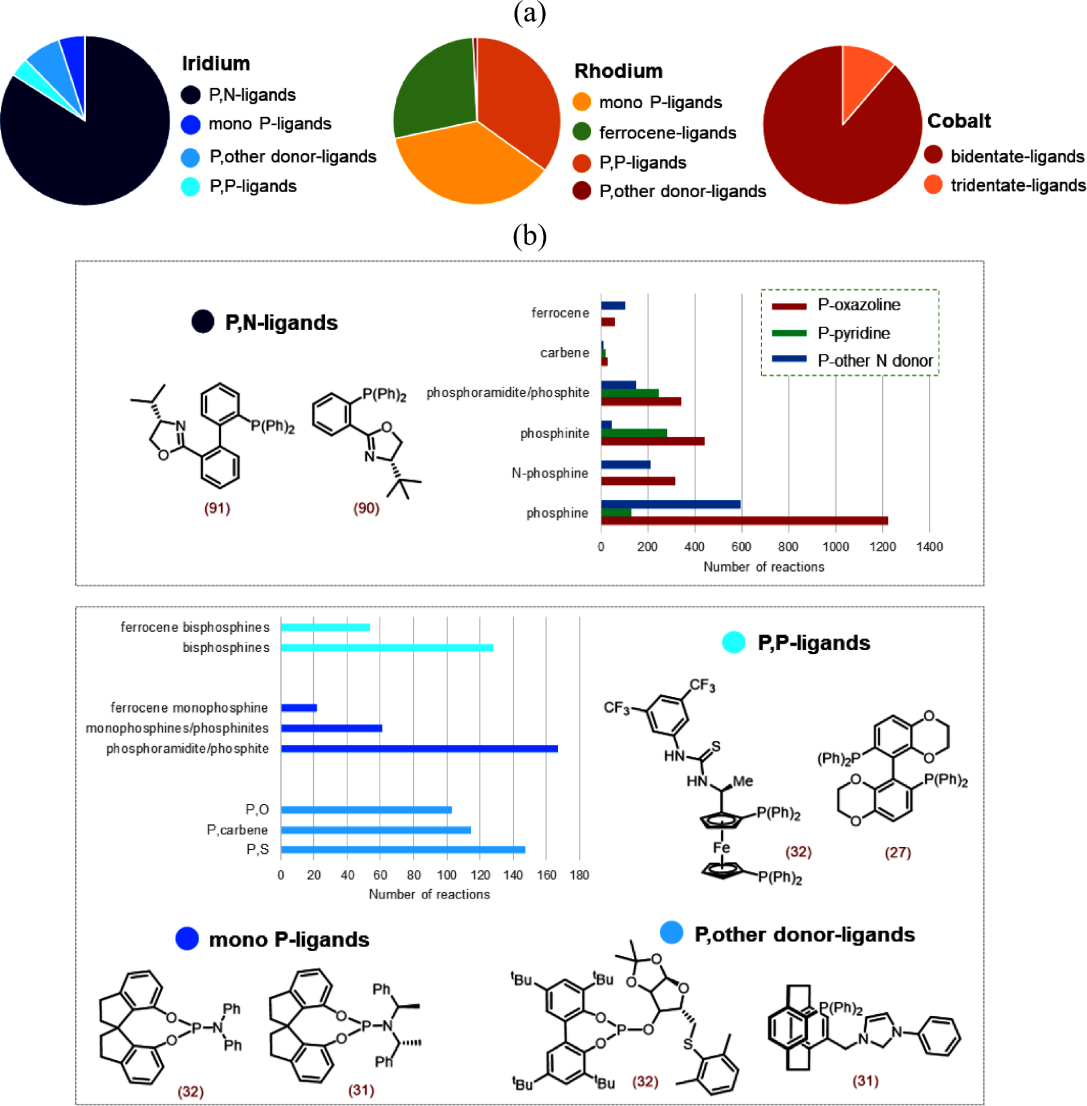
(a) Distribution of the types of ligands used with Ir, Rh, and Co metal catalysts in the AHO. (b) A detailed analysis of the nature of ligands investigated with Ir catalytic systems in asymmetric hydrogenation. The two most used ligands of each type are shown along with the number of reactions (in parentheses).

In this section, we analyze the type of ligands used with Ir-, Rh-, and Co-catalyzed AHO. The classification of ligands into various categories and their respective distributions for all three metal catalysts are shown in Figure 4a. With 85% of the reported reactions, the chiral bidentate P,N ligands dominate the Ir-catalyzed AHO.^49^ On the other hand, there are relatively fewer examples of other bidentate P,P and P,other donor atom ligands, along with monodentate phosphorus ligands. In sharp contrast, P and N ligands are rarely used with Rh catalysts. However, monodentate and bidentate phosphorus ligands have been used extensively in Rh-catalyzed AHO reactions (Figure 4a).^50^ Furthermore, the chiral ferrocene-derived monodentate and bidentate phosphorus ligands are also well-explored. The type of ligands developed for Co catalysts more closely resemble Rh, with over 65% reactions using bidentate phosphorus ligands. Unlike Ir and Rh, chiral tridentate ligands are also employed with Co catalysts.^51^ Each of the ligand types is further categorized into different classes as described below.

First, the class of ligands utilized in Ir-catalyzed AHO is studied. As discussed above, P,N ligands bearing P and N donor atoms are the most widely used (Figure 4a). The scope of these ligands has been extended over the years by modifying the identity of the P and N donor groups. P,N ligands with an oxazoline N donor and phosphine-type P donor are the most common, with roughly 25% of the reactions (Figure 4b). Other P donor group analogues, such as *N*-phosphine, phosphinite, phosphite, and phosphoramidite, are also used with an oxazoline N donor, which together constitute about 22% of the reactions. All of these P donor groups in combination with different N donor groups (replacing oxazoline) such as pyridine, thiazole, imidazole, etc., account for over 35% of the reported reactions. Moreover, there are some examples of ligands with a phosphine group being replaced by a carbene moiety and chiral ferrocene P,N ligands.

There are a few instances of the use of chiral non-N-containing ligands for Ir-catalyzed asymmetric hydrogenation, obtained by changing the nature of the N donor atom in the popular P,N ligands. Some of the examples include P–carbene, P–S, and P–O ligands, where the P donor group is usually a phosphine or a phosphite ligand (Figure 4b). Monodentate phosphorus ligands, such as phosphines, phosphites, phosphoramidites, etc., have also been explored. Some examples of bisphosphine and ferrocenyl bisphosphine ligands are also present. The structures of the two most common ligands with the maximum number of reactions are shown in Figure 4b for each ligand type.

**Figure 5:**
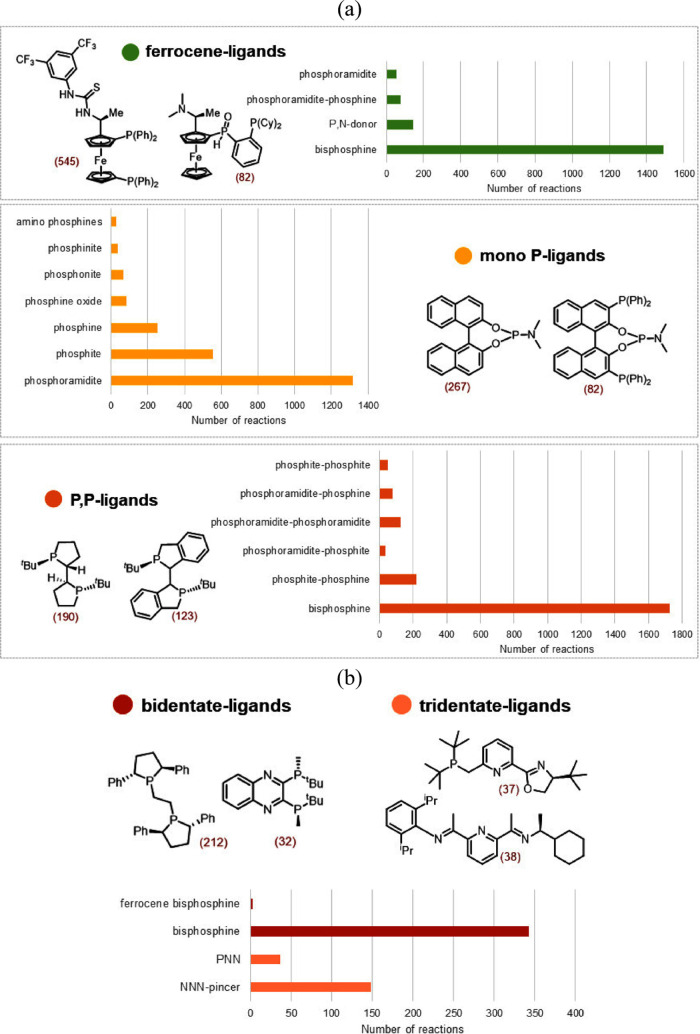
A detailed analysis of the nature of ligands investigated with (a) rhodium and (b) cobalt catalytic systems in asymmetric hydrogenation. The two most used ligands of each type are shown along with the number of reactions (in parentheses).

The ligands used in Rh-catalyzed AHO reactions are considerably different from those used in Ir catalytic systems. Monodentate phosphorus ligands such as phosphines, phosphites, and phosphoramidites are the most common, followed by a few examples of phosphine oxides, phosphonites, and phosphinites (Figure 5a). Monodentate phosphoramidites have been used more frequently with over 20% reactions. Bidentate phosphorus ligands are another class of popular ligands for asymmetric hydrogenation using Rh catalysts. Among different types of P,P ligands, bisphosphines are utilized predominantly, with around 27% of all reported reactions (Figure 5a). Other P,P ligands, although relatively infrequent, include phosphine–phosphite, phosphine–phosphoramidite, phosphite–phosphoramidite, etc. Ferrocene-based mono- and bidentate ligands form another one-third of the ligands used in Rh-catalyzed asymmetric hydrogenation reactions. Ferrocene-based bisphosphines dominate with over 23% of the reactions, followed by a fewer number of phosphoramidite–phosphine and P,N ligands. Analogous to Rh, the ligands used in Co-catalyzed asymmetric hydrogenation consist primarily of chiral bidentate phosphines (Figure 5b). Unlike Ir and Rh, tridentate ligands have also been explored with Co metal. NNN-type ligands, for instance, bis(imino)pyridine, are prevalent, with about 28% reactions. Besides, a small number of reactions with PNN-type ligands such as phosphine–pyridine–oxazoline are also present. The ligands with the maximum number of reactions used in Rh- and Co-catalyzed AHO reactions are shown in Figure 5. We also labeled each ligand type for all three metal catalysts based on the median enantioselectivity (Figure S5). A detailed comparison of the trends is described in the Supporting Information (section 5).

**Figure 6:**
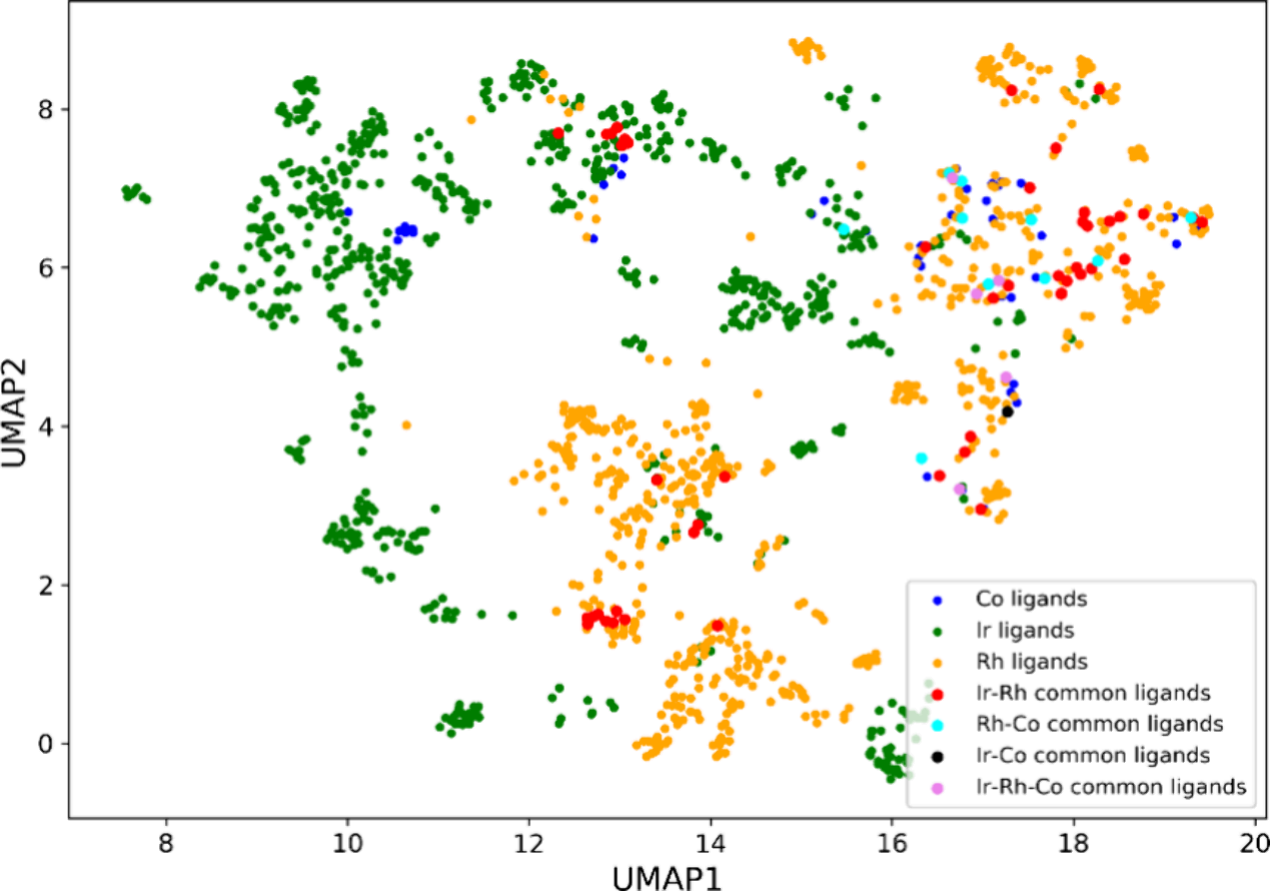
UMAP plot of the chemical space of ligands used in Ir-, Rh-, and Co-catalyzed asymmetric hydrogenation. The *x*- and *y*-axes correspond to the two UMAP components obtained after dimensionality reduction.

The chemical space covered by the ligands utilized in asymmetric hydrogenation catalyzed by all three metal catalysts is visualized by using a UMAP plot. Figure 6 shows the UMAP plot for the ligands used with Ir (shown in green), Rh (shown in orange), Co (shown in blue), and the ligands common among the metals (see Figure S3 for individual UMAP plots). In contrast to the UMAP plot for olefins (Figure 3), there is not much overlap in the regions occupied by the ligands used in Ir- and Rh-catalyzed AHO reactions. The chemical space covered by the Ir ligands is well-separated from that of the Rh ligands. There are relatively fewer ligands common to both Ir and Rh, occupying mainly the chemical space of Rh ligands (Table S5). The ligands used with Co share most of the chemical space with Rh, while some are found to be closer to Ir as well (Figure 6). Further, Co has more ligands in common with Rh as opposed to Ir. These insights can provide directions to expand the ligand space for cobalt catalysts. Additionally, we visualized the chemical space covered by the ligands based on their average enantioselectivity values (Figure S7). A greater variation in the enantioselectivity is noted with the ligand space compared to the olefin chemical space. A comparison of the UMAP plot with another nonlinear dimensionality reduction technique is also carried out. For this purpose, the t-distributed stochastic neighbor embedding (t-SNE) algorithm is used to visualize the olefin and ligand chemical space (Figure S8). It is noted that the regions of chemical space occupied by various olefins and ligands are similar for both t-SNE (Figure S8) and UMAP (Figures 3 and 6) plots.

In summary, various key points can be noted about the type of ligands commonly used with Ir, Rh, and Co catalysts from data-driven analysis. The Ir-catalyzed AHO reactions are dominated by P,N ligands, where the phosphine P donor and oxazoline N donor have been widely used. In contrast to Ir, the asymmetric hydrogenation with Rh catalysts is primarily with mono- and bidentate phosphorus ligands, among which phosphoramidites and bisphosphines are the most prevalent, with 70% reactions. Although the ligand space explored in Co-catalyzed asymmetric hydrogenation is relatively small, it more closely resembles Rh ligands and is largely composed of bisphosphines.

### Reaction Conditions

**Figure 7:**
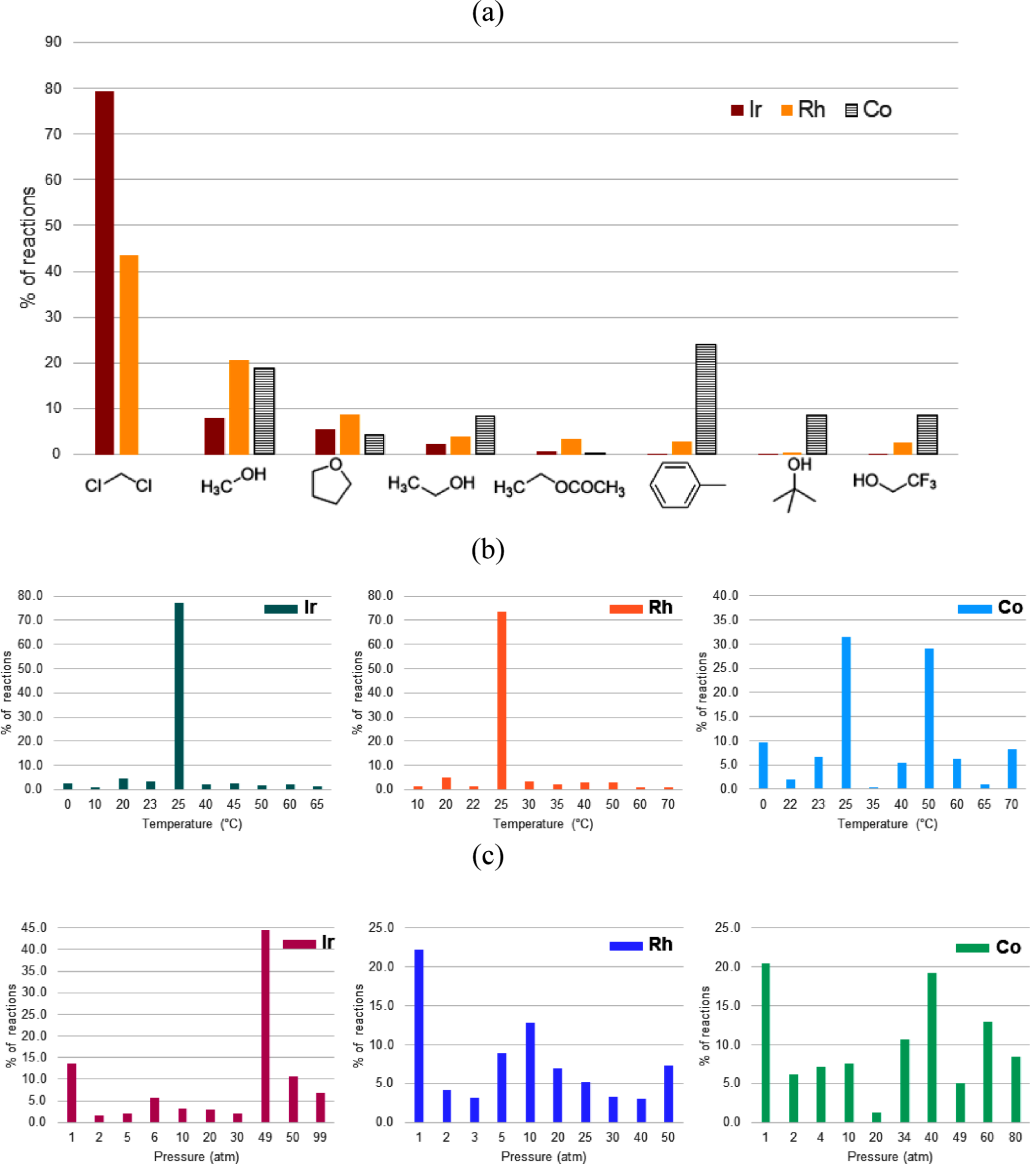
(a) Solvents and ranges of (b) temperature and (c) pressure mostly used in the Ir-, Rh-, and Co-catalyzed AHO.

The identification of the reaction conditions suitable for a given substrate is an important problem in chemical synthesis. In this section, we analyze the choice of solvents and the range of temperatures and pressures in the AHO catalyzed by all three metal catalysts. The solvents with the maximum number of reactions are shown in Figure 7a. The polar noncoordinating dichloromethane (DCM) is the most popular solvent for Ir catalysts, with around 80% reactions. Similarly, more than 40% of the Rh-catalyzed AHO reactions use DCM as the solvent. The other 60% of the reactions are reported primarily with polar coordinating solvents such as methanol, tetrahydrofuran, ethanol, etc. While Ir- and Rh-catalyzed asymmetric hydrogenation takes place almost entirely in polar solvents, 25% of the reactions with Co use the nonpolar solvent toluene. Alcohol solvents such as ethanol, methanol, tert-butanol, trifluoroethanol, and so on are also frequently utilized in Co-catalyzed AHO reactions (Figure 7a). More importantly, environmentally unfriendly DCM is not a solvent of choice for reactions with Co catalysts as opposed to that of Ir.

A plot of the top 10 temperature and pressure values used with Ir, Rh, and Co catalysts in asymmetric hydrogenation is shown in Figure 7b,c, respectively. More than 70% of the reactions reported with Ir and Rh catalysts occur at room temperature, whereas it is only 30% for Co catalysts. Another 30% of the reactions with Co are performed at 50 °C (Figure 7b). Regarding pressure, over 60% of the reactions are carried out at 49–50 atm pressure with Ir catalysts. In contrast, reactions with Rh catalysts are reported at pressures lower than 50 atm. This implies that the asymmetric hydrogenation with Rh usually takes place at a pressure lower than that with Ir. The distribution of pressure in the case of reactions with Co, to a certain extent, is a mix of both Ir and Rh, where both low and high pressures are used significantly (Figure 7c).

### Selectivity Trends with Olefin–Ligand Combinations

**Figure 8:**
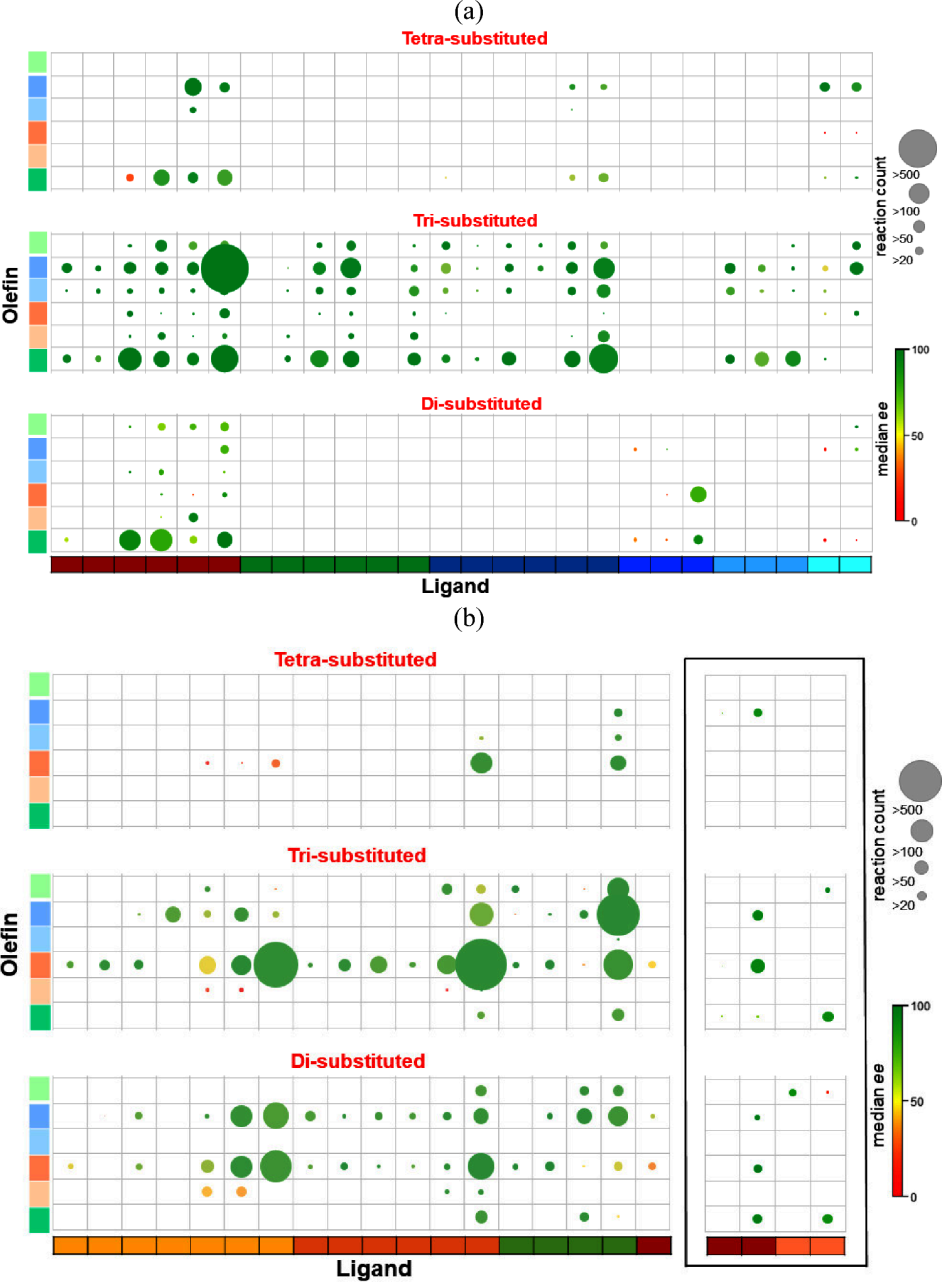
Plots of olefin–ligand combinations for (a) Ir, (b) Rh, and Co (enclosed in a black box)-catalyzed asymmetric hydrogenation. The y-axis corresponds to olefin type where each color displays the identity of olefin, as shown in Figure 2. The x-axis represents the type of ligand. The identity of ligand (shown using various colors) can be found in Figures 4b, 5a, and 5b for Ir, Rh, and Co respectively. The circle size corresponds to the number of reactions. The color corresponds to the median enantioselectivity of all reactions in the given category.

In this section, we discuss the olefin–ligand combinations used in the asymmetric hydrogenation catalyzed by Ir, Rh, and Co metal catalysts (Figure 8). The data is analyzed in terms of the median enantioselectivity and the number of reactions corresponding to a particular olefin–ligand combination. The ligand combination with di-, tri-, and tetrasubstituted olefins is studied separately for a better understanding. The large number of empty boxes in Figure 8 reveals that the olefin–ligand combinations utilized in asymmetric hydrogenation are very sparse. There are only a few types of ligands that have been used with all olefin types. The dominance of green circles again indicates the bias in the literature reporting reactions with higher selectivities (Figure 1b).

For the Ir-catalyzed asymmetric hydrogenation of trisubstituted olefins, reactions using most of the olefin–ligand combinations are present, with monodentate phosphorus ligands being an exception. Although all P,N-type ligands exhibit performance comparable to those of aryl- and alkyl-substituted alkenes, the ligands with the maximum number of reactions include phosphite/phosphoramidite–oxazoline, phosphine–oxazoline, and phosphine–other-N-donor (Figure 8a). Phosphine–oxazoline is also the most used ligand with α,β-unsaturated carbonyls. In the case of disubstituted olefins, only the aryl- and alkyl-substituted alkenes with the P-oxazoline type of ligands are majorly present with phosphite/phosphoramidite, phosphinite, and phosphine as the P donors. All other combinations have a smaller number of reactions with a relatively lower median enantioselectivity. Similar to disubstituted olefins, only a few olefin–ligand combinations have been reported for tetrasubstituted olefins (Figure 8a). The *N*-phosphine–oxazoline type of ligand has a relatively higher median %ee with both aryl- and alkyl-substituted alkenes and α,β-unsaturated carbonyls. The phosphinite/phosphine–oxazoline ligand has, in general, a lower %ee with aryl- and alkyl-substituted alkenes. It is quite evident from Figure 8a that phosphine–oxazoline has been used with most of the olefins, exhibiting good enantioselectivities, thereby making it a ligand of choice in Ir-catalyzed AHO reactions. The top 10 phosphine–oxazoline ligands are shown in the Supporting Information (Table S2).

The olefin–ligand combination for the asymmetric hydrogenation of trisubstituted olefins using a Rh catalyst is shown in Figure 8b. Almost every ligand has been used in combination with enamides and enamines. However, reactions with three ligands, i.e., bisphosphines, phosphoramidites, and ferrocenyl bisphosphines, stand out compared to other ligands. Reactions with α,β-unsaturated carbonyls are primarily reported with ferrocenyl bisphosphines with a high %ee. Unlike enamides, bisphosphines and phosphoramidites have been used less frequently with α,β-unsaturated carbonyls and with reduced enantioselectivities. Alkenes bearing other heteroatoms also have high %ee values with ferrocenyl bisphosphines. The olefin–ligand combination for disubstituted olefins has been comparatively better explored with Rh than with Ir (Figure 8b). For disubstituted olefins, enamides have much fewer reactions and a lower median %ee with ferrocenyl bisphosphines. On the other hand, bisphosphines and phosphoramidites have the maximum number of reactions and provide a higher %ee with enamides. In addition, the monodentate phosphite ligand provides good performance with enamides and α,β-unsaturated carbonyl also. In the case of tetrasubstituted olefins, the reaction of enamides with bisphosphines and ferrocenyl bisphosphines is the most prominent. However, the success is limited for reactions with monodentate phosphorus ligands. The top 10 of the three most successful ligands in Rh-catalyzed AHO reactions, namely, bisphosphine, phosphoramidite, and ferrocenyl bisphosphine, are shown in the Supporting Information (Table S3).

Co-catalyzed AHO has witnessed progress only in recent years. Therefore, the diversity of olefins and ligands explored using Co catalysts is limited (enclosed in a black-colored box in Figure 8b). Bisphosphines have been reported mainly in combination with enamides and α,β-unsaturated carbonyls. On the other hand, aryl- and alkyl-substituted alkenes have more reactions with tridentate NNN-type ligands. The top 5 ligands with Co metal are shown in the Supporting Information (Table S4). Finally, certain olefin–ligand combinations can be identified from Figure 8 for each of the metal catalysts that exhibit high enantioselectivities but have not been explored much. For instance, in the case of reactions with Ir catalysts, phosphite and phosphoramidite ligands with disubstituted aryl- and alkyl-substituted alkenes and ferrocenyl bisphosphines with tetrasubstituted α,β-unsaturated carbonyls show potential. Similarly, for Rh-catalyzed AHO reactions, ferrocene-based P,N-type ligands with disubstituted alkenes bearing other heteroatoms and aryl- and alkyl-substituted alkenes have shown promise.

## Conclusions

In this work, we analyzed the reaction space of transition-metal-catalyzed AHO from a data-driven perspective. Iridium and rhodium metal catalysts have traditionally been used for this type of reaction, whereas the cobalt catalyst is a reasonably new entrant. The Co-catalyzed asymmetric hydrogenation has become popular in recent years due to its unique reactivity and the growing need for the use of earth-abundant transition metals. The reaction data on asymmetric hydrogenation for all three metal catalysts are studied simultaneously to understand the similarities and differences in their reactivity patterns. For this purpose, olefins and ligands are first classified into various chemically relevant categories. The types of olefins and ligands suitable for a given metal catalyst are examined. For instance, the unfunctionalized and/or minimally functionalized olefins with P,N-type ligands are found to be a good combination for Ir catalysts. However, functionalized olefins with bisphosphines and phosphoramidites as ligands are well-explored using Rh catalysts. The types of olefins used with Co catalysts are similar to those of the other two metals. On the other hand, bisphosphines are the popular ligands with Co, which more closely resemble the ligands used with Rh metal. Additionally, the reaction conditions, including solvent, temperature, and pressure, are also evaluated. There are only a few solvents that are used in most of the reactions. For example, 80% of the Ir-catalyzed asymmetric hydrogenation has been carried out in DCM as the solvent. Similarly, there is a significant imbalance in the diversity of olefins and ligands. Although various olefins and ligands have been explored in asymmetric hydrogenation, there are only a few frequently used olefin–ligand combinations that constitute the majority of the reported reactions. Also, the reaction performance is found to be highly skewed toward high enantioselectivities. To sum up, this study highlights the data-inherent bias of the literature-mined data sets in terms of the diversity of substrates, ligands, reaction conditions, and performance. This presents a challenge for machine-learning models to provide meaningful predictions as they may capture only the literature trends. Therefore, we emphasize the need for exploring a diverse range of substrates, ligands, and reaction conditions, along with prioritizing the reporting of failed or low-yielding reactions. This would enable practitioners to realize the benefits of machine-learning and other data-driven methods for reaction development.

## Data Availability

The data underlying this study are available in the published article and its Supporting Information.

## Supporting Information Available

Diversity of olefins and ligands, median enantioselectivity of different olefin and ligand types, visualization of olefin and ligand chemical space (Tables S1–S5 and Figures S1–S8) (PDF)
SMILES of substrates, ligands, solvents, additives, and products along with the experimental variables of pressure, temperature, and catalyst loading (XLSX)

## References

[ref1] Behera P.; Ramakrishna D. S.; Chandrasekhar M. M.; Kothakapu S. R. A concise review on recent advances in catalytic asymmetric hydrogenation. Chirality 2023, 35, 477–497. 10.1002/chir.23559. 36960690

[ref2] Etayo P.; Vidal-Ferran A. Rhodium-catalyzed asymmetric hydrogenation as a valuable synthetic tool for the preparation of chiral drugs. Chem. Soc. Rev. 2013, 42, 728–754. 10.1039/C2CS35410A. 23132556

[ref3] Seo C. S. G.; Morris R. H. Catalytic homogeneous asymmetric hydrogenation: successes and opportunities. Organometallics 2019, 38, 47–65. 10.1021/acs.organomet.8b00774.

[ref4] Andersson P. G.; Margarita C. Evolution and prospects of the asymmetric hydrogenation of unfunctionalized olefins. J. Am. Chem. Soc. 2017, 139, 1346–1356. 10.1021/jacs.6b10690. 28064490

[ref5] Massaro L.; Zheng J.; Margarita C.; Andersson P. G. Enantioconvergent and enantiodivergent catalytic hydrogenation of isomeric olefins. Chem. Soc. Rev. 2020, 49, 2504–2522. 10.1039/C9CS00138G. 32202283

[ref6] Genêt J.-P. Modern Reduction Methods; Andersson P. G., Munslow I. J., Eds.; Wiley-VCH: Weinheim, 2008.

[ref7] Verendel J. J.; Pamies O.; Dieguez M.; Andersson P. G. Asymmetric hydrogenation of olefins using chiral crabtree-type catalysts: scope and limitations. Chem. Rev. 2014, 114, 2130–2169. 10.1021/cr400037u. 24568181

[ref8] Cabre A.; Verdaguer X.; Riera A. Recent advances in the enantioselective synthesis of chiral amines via transition metal-catalyzed asymmetric hydrogenation. Chem. Rev. 2022, 122, 269–339. 10.1021/acs.chemrev.1c00496. 34677059 PMC9998038

[ref9] Luckemeier L.; Pierau M.; Glorius F. Asymmetric arene hydrogenation: towards sustainability and application. Chem. Soc. Rev. 2023, 52, 4996–5012. 10.1039/D3CS00329A. 37427715 PMC10389296

[ref10] Peters B. B. C.; Zheng J.; Birke N.; Singh T.; Andersson P. G. Iridium-catalyzed enantioconvergent hydrogenation of trisubstituted olefins. Nat. Commun. 2022, 13, 361 10.1038/s41467-022-28003-6. 35042913 PMC8766446

[ref11] Wu X.; Su Y.; Zi G.; Ye W.; Hou G. Rh-catalyzed asymmetric hydrogenation of alpha-substituted alkenyl sulfones: highly chemo- and enantioselective access to chiral sulfones. Org. Lett. 2023, 25, 6858–6862. 10.1021/acs.orglett.3c02414. 37703279

[ref12] Chakrabortty S.; Konieczny K.; Moritz J.-O.; Zheng S.; et al. Rh-catalyzed enantioselective hydrogenation of di- and tri-substituted enamides enabled by easily tunable P-stereogenic N-phosphinyl phosphoramidite ligands. ACS Catal. 2023, 13, 12030–12040. 10.1021/acscatal.3c03336.

[ref13] Kraft S.; Ryan K.; Kargbo R. B. Recent advances in asymmetric hydrogenation of tetrasubstituted olefins. J. Am. Chem. Soc. 2017, 139, 11630–11641. 10.1021/jacs.7b07188. 28800391

[ref14] Wen J.; Wang F.; Zhang X. Asymmetric hydrogenation catalyzed by first-row transition metal complexes. Chem. Soc. Rev. 2021, 50, 3211–3237. 10.1039/D0CS00082E. 33480901

[ref15] Morris R.; Chirik P. Getting down to earth: the renaissance of catalysis with abundant metals. Acc. Chem. Res. 2015, 48, 2495 10.1021/acs.accounts.5b00385. 26370392

[ref16] Ye F.; Xu Z.; Xu L.-W. The discovery of multifunctional chiral P ligands for the catalytic construction of quaternary carbon/silicon and multiple stereogenic centers. Acc. Chem. Res. 2021, 54, 452–470. 10.1021/acs.accounts.0c00740. 33375791

[ref17] Xu G.; Senanayake C. H.; Tang W. P-chiral phosphorus ligands based on a 2,3-dihydrobenzo[*d*][1,3]oxaphosphole motif for asymmetric catalysis. Acc. Chem. Res. 2019, 52, 1101–1112. 10.1021/acs.accounts.9b00029. 30848882

[ref18] Gensch T.; dos Passos Gomes G.; Friederich P.; Peters E.; Gaudin T.; Pollice R.; Jorner K.; Nigam A.; Lindner-D’Addario M.; Sigman M. S.; Aspuru-Guzik A. A comprehensive discovery platform for organophosphorous ligands for catalysis. J. Am. Chem. Soc. 2022, 144, 1205–1217. 10.1021/jacs.1c09718. 35020383

[ref19] Gromski P. S.; Henson A. B.; Granda J. M.; Cronin L. How to explore chemical space using algorithms and automation. Nat. Rev. Chem. 2019, 3, 119–128. 10.1038/s41570-018-0066-y.

[ref20] Filipa de Almeida A.; Moreira R.; Rodrigues T. Synthetic organic chemistry driven by artificial intelligence. Nat. Rev. Chem. 2019, 3, 589–604. 10.1038/s41570-019-0124-0.

[ref21] Tu Z.; Stuyver T.; Coley C. W. Predictive chemistry: machine learning for reaction deployment, reaction development, and reaction discovery. Chem. Sci. 2023, 14, 226–244. 10.1039/D2SC05089G. 36743887 PMC9811563

[ref22] Liu H.-W.; He P.; Li W.-T.; Sun W.; Shi K.; Wang Y.-Q.; Mo Q.-K.; Zhang X.-Y.; Zhu S.-F. Catalyst-oriented design based on elementary reactions (CODER) fir triarylamine synthesis. Angew. Chem., Int. Ed. 2023, 62, e202309111 10.1002/anie.202309111. 37698233

[ref23] Zhang Z.-J.; Li S.-W.; Oliveira J. C. A.; Li Y.; Chen X.; Zhang S.-Q.; Xu L.-C.; Rogge T.; Hong X.; Ackermann L. Data-driven design of new chiral carboxylic acid for construction of indoles with C-central and C-N axial chirality via cobalt catalysis. Nat. Commun. 2023, 14, 3149 10.1038/s41467-023-38872-0. 37258542 PMC10232535

[ref24] Karl T. M.; Bouayad-Gervais S.; Hueffel J. A.; Sperger T.; Wellig S.; Kaldas S. J.; Dabranskaya U.; Ward J. S.; Rissanen K.; Tizzard G. J.; Schoenebeck F. Machine Learning-Guided Development of Trialkylphosphine Ni (I) Dimers and Applications in Site-Selective Catalysis. J. Am. Chem. Soc. 2023, 145, 15414–15424. 10.1021/jacs.3c03403. 37411044

[ref25] Caldeweyher E.; Elkin M.; Gheibi G.; Johansson M.; Sköld C.; Norrby P.-O.; Hartwig J. F. Hybrid machine learning approach to predict the site selectivity of iridium-catalyzed arene borylation. J. Am. Chem. Soc. 2023, 145, 17367–17376. 10.1021/jacs.3c04986. 37523755 PMC11723321

[ref26] Singh S.; Sunoj R. B. Molecular machine learning for chemical catalysis: prospects and challenges. Acc. Chem. Res. 2023, 56, 402–412. 10.1021/acs.accounts.2c00801. 36715248

[ref27] Kariofillis S. K.; Jiang S.; Zuranski A. M.; Gandhi S. S.; Alvarado J. I. M.; Doyle A. G. Using data science to guide aryl bromide substrate scope analysis in a Ni/photoredox-catalyzed cross-coupling with acetals as alcohol-derived radical sources. J. Am. Chem. Soc. 2022, 144, 1045–1055. 10.1021/jacs.1c12203. 34985904 PMC8810294

[ref28] Rana D.; Pfluger P. M.; Holter N. P.; Tan G.; Glorius F. Standardizing substrate selection: a strategy toward unbiased evaluation of reaction generality. ACS Cent. Sci. 2024, 10, 899–906. 10.1021/acscentsci.3c01638. 38680564 PMC11046462

[ref29] Kutchukian P. S.; Dropinski J. F.; Dykstra K. D.; Li B.; DiRocco D. A.; Streckfuss E. C.; Campeau L.-C.; Cernak T.; Vachal P.; Davies I. W.; Krska S. W.; Dreher S. D. Chemistry informer libraries: a chemoinformatics enabled approach to evaluate and advance synthetic methods. Chem. Sci. 2016, 7, 2604–2613. 10.1039/C5SC04751J. 28660032 PMC5477042

[ref30] Beker W.; Roszak R.; Wolos A.; Angello N. H.; Rathore V.; Burke M. D.; Grzybowski B. A. Machine learning may sometimes simply capture literature popularity trends: a case study of heterocyclic Suzuki-Miyaura coupling. J. Am. Chem. Soc. 2022, 144, 4819–4827. 10.1021/jacs.1c12005. 35258973 PMC8949728

[ref31] Strieth-Kalthoff F.; Sandfort F.; Kuhnemund M.; Schafer F. R.; Kuchen H.; Glorius F. Machine learning for chemical reactivity: the importance of failed experiments. Angew. Chem., Int. Ed. 2022, 61, e202204647 10.1002/anie.202204647. 35512117

[ref32] Betinol I. O.; Lai J.; Thakur S.; Reid J. P. A data-driven workflow for assigning and predicting generality in asymmetric catalysis. J. Am. Chem. Soc. 2023, 145, 12870–12883. 10.1021/jacs.3c03989. 37266999

[ref33] Rinehart N. I.; Saunthwal R. K.; Wellauer J.; Zahrt A. F.; Schlemper L.; Shved A. S.; Bigler R.; Fantasia S.; Denmark S. E. A machine-learning tool to predict substrate-adaptive conditions for Pd-catalyzed C-N couplings. Science 2023, 381, 965–972. 10.1126/science.adg2114. 37651532

[ref34] Pitzer L.; Schafers F.; Glorius F. Rapid assessment of the reaction-condition-based sensitivity of chemical transformations. Angew. Chem., Int. Ed. 2019, 58, 8572–8576. 10.1002/anie.201901935. 30932282

[ref35] Schrader M. L.; Schafer F. R.; Schafers F.; Glorius F. Bridging the information gap in organic chemical reactions. Nat. Chem. 2024, 16, 491–498. 10.1038/s41557-024-01470-8. 38548884

[ref36] Fitzner M.; Wuitschik G.; Koller R. J.; Adam J.-M.; Schindler T.; Reymond J.-L. What can reaction databases teach us about Buchwald-Hartwig cross-couplings?. Chem. Sci. 2020, 11, 13085 10.1039/D0SC04074F. 34476050 PMC8378852

[ref37] Pereira A.; Albornoz C.; Trofymchuk O. S. Data-driven analysis of reactions catalyzed by [CoCp*(CO)I_2_]. Organometallics 2022, 41, 1158–1166. 10.1021/acs.organomet.2c00051.

[ref38] Raghavan P.; Haas B. C.; Ruos M. E.; Schleinitz J.; Doyle A. G.; Reisman S. E.; Sigman M. S.; Coley C. W. Dataset design for building models of chemical reactivity. ACS Cent. Sci. 2023, 9, 2196 10.1021/acscentsci.3c01163. 38161380 PMC10755851

[ref39] Dotson J. J.; van Dijk L.; Timmerman J. C.; Grosslight S.; Walroth R. C.; Gosselin F.; Püntener K.; Mack K. A.; Sigman M. S. Data-driven multi-objective optimization tactics for catalytic asymmetric reactions using bisphosphine ligands. J. Am. Chem. Soc. 2023, 145, 110–121. 10.1021/jacs.2c08513. 36574729 PMC10194998

[ref40] van Dijk L.; Haas B. C.; Lim N.-K.; Clagg K.; Dotson J. J.; Treacy S. M.; Piechowicz K. A.; Roytman V. A.; Zhang H.; Toste F. D.; Miller S. J.; Gosselin F.; Sigman M. S. Data Science-Enabled Palladium-Catalyzed Enantioselective Aryl-Carbonylation of Sulfonimidamides. J. Am. Chem. Soc. 2023, 145, 20959–20967. 10.1021/jacs.3c06674. 37656964

[ref41] Xu Li-C; Zhang S.-Q.; Li X.; Tang M.-J.; Xie P.-P.; Hong X. Towards data-driven design of asymmetric hydrogenation of olefins: database and hierarchical learning. Angew. Chem., Int. Ed. 2021, 60, 22804–22811. 10.1002/anie.202106880. 34370892

[ref42] The full dataset is available at http://asymcatml.net.

[ref43] Weininger D. SMILES-A language for molecules and reactions. Handbook of Chemoinformatics 2003, 80–102. 10.1002/9783527618279.ch5.

[ref44] Schleinitz J.; Langevin M.; Smail Y.; Wehnert B.; Grimaud L.; Vuilleumier R. Machine learning yield prediction from NiCOlit, a small-size literature data set of nickel catalyzed C-O couplings. J. Am. Chem. Soc. 2022, 144, 14722–14730. 10.1021/jacs.2c05302. 35939717

[ref45] Lu J.; Leitch D. C. Organopalladium catalysis as a proving ground for data-rich approaches to reaction development and quantitative predictions. ACS Catal. 2023, 13, 15691–15707. 10.1021/acscatal.3c03864.

[ref46] Charvillat T.; Bernardelli P.; Daumas M.; Pannecoucke X.; Ferey V.; Besset T. Hydrogenation of fluorinated molecules: an overview. Chem. Soc. Rev. 2021, 50, 8178–8192. 10.1039/D0CS00736F. 34060550

[ref47] McInnes L.; Healy J.; Melville J. UMAP: uniform manifold approximation and projection for dimension reduction. arXiv 2020, 10.48550/arXiv.1802.03426.

[ref48] Durant J. L.; Leland B. A.; Henry D. R.; Nourse J. G. Reoptimization of MDL keys for use in drug discovery. J. Chem. Inf. Comput. Sci. 2002, 42, 1273–1280. 10.1021/ci010132r. 12444722

[ref49] Peters B. B. C.; Andersson P. G. The implications of the bronsted acidic properties of crabtree-type catalysts in the asymmetric hydrogenation of olefins. J. Am. Chem. Soc. 2022, 144, 16252–16261. 10.1021/jacs.2c07023. 36044252 PMC9479089

[ref50] Sen A.; Chikkali S. H. C_1_-symmetric diphosphorous ligands in metal-catalyzed asymmetric hydrogenation to prepare chiral compounds. Org. Biomol. Chem. 2021, 19, 9095 10.1039/D1OB01207J. 34617539

[ref51] Wang H.; Wen J.; Zhang X. Chiral tridentate ligands in transition metal-catalyzed asymmetric hydrogenation. Chem. Rev. 2021, 121, 7530–7567. 10.1021/acs.chemrev.1c00075. 34014646

