# Supplementary material for: Data-Driven Insights into the Transition-Metal-Catalyzed Asymmetric Hydrogenation of Olefins
Source: J Org Chem. 2024 Aug 16;89(17):12467–78. doi: 10.1021/acs.joc.4c01396 (PMC11382158; doi:10.1021/acs.joc.4c01396)
Supplement: Supplementary file 1 — jo4c01396_si_001.pdf [file jo4c01396_si_001.pdf]

Supporting Information for

Data-Driven Insights on Transition Metal-Catalyzed Asymmetric Hydrogenation of Olefins

Sukriti Singh* and José Miguel Hernández-Lobato*

Department of Engineering, University of Cambridge, Cambridge CB2 1PZ, United Kingdom

Email: sukriti243@gmail.com; jmh233@cam.ac.uk

	Table of Contents	Page
1.	Diversity of olefins	S3
2.	Median enantioselectivity of different olefin types	S4
3.	Visualization of olefin chemical space	S5
4.	Diversity of ligands	S6
5.	Median enantioselectivity of different ligand types	S11
6.	Visualization of ligand chemical space	S13
7.	Visualization of olefin and ligand chemical space using t-SNE	S14

1. Diversity of olefins

The five most-used di-, tri-, and tetra-substituted olefins in Ir-, Rh-, and Co-catalyzed asymmetric hydrogenation is shown in Figure S1.

(a)

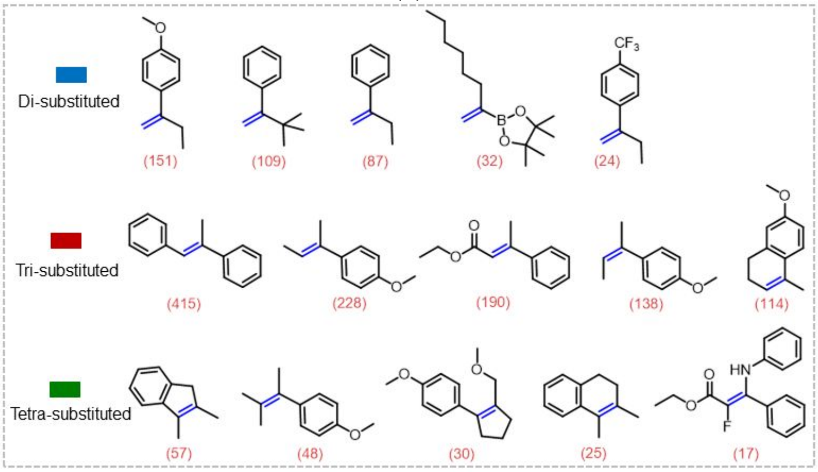

(b)

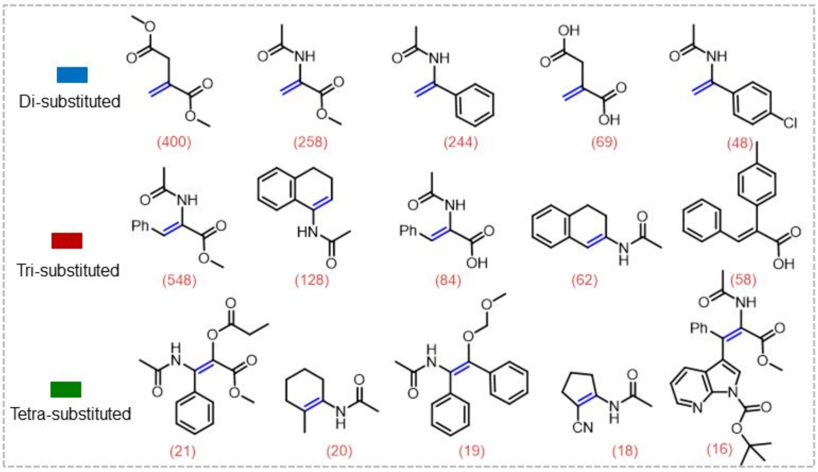

(c)

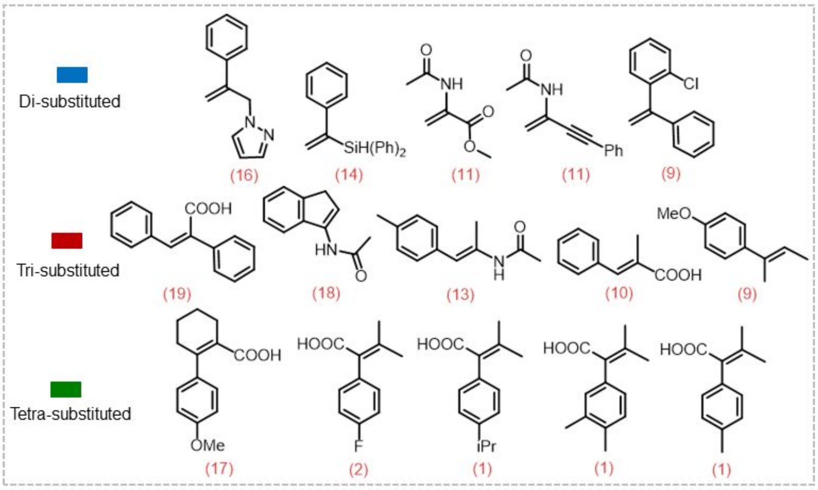

Figure S1. Top-5 di-, tri-, and tetrasubstituted olefins in (a) iridium, (b) rhodium, and (c) cobalt-catalyzed asymmetric hydrogenation. The number of reactions for a given olefin is shown in parentheses.

The number of unique olefins for each metal catalyst as well as the common olefins are shown in Table S1. The number of olefins common among different metal catalysts is limited. The type of olefins used with Ir and Rh catalysts that are common to Co includes aryl- and alkyl-substituted alkenes, enamides and α,β-unsaturated carbonyls. The most common olefin types between Ir and Rh are α,β-unsaturated carbonyls and alkenes bearing other heteroatoms like B, P, S, etc.

Table S1. The Number of Olefins Corresponding to Each Metal Catalyst

S. No.	Olefin type	Number of olefins
1.	Unique olefins with Ir	1026
2.	Unique olefins with Rh	1240
3.	Unique olefins with Co	288
4.	Common olefins with Ir and Rh	102
5.	Common olefins with Rh and Co	25
6.	Common olefins with Ir and Co	34
7.	Common olefins with Ir, Rh, and Co	19

2. Median enantioselectivity of different olefin types

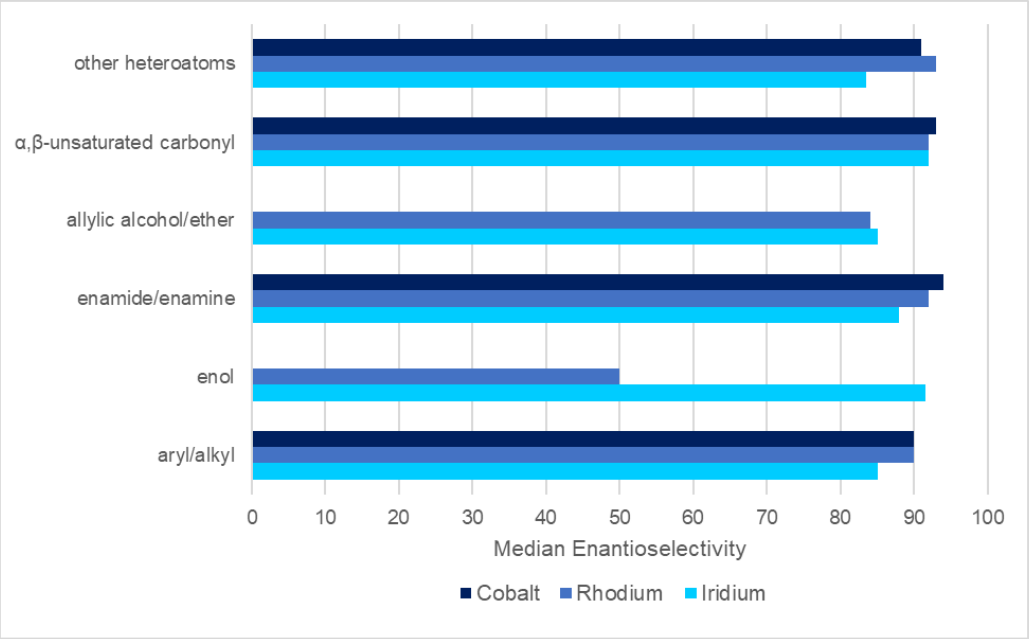

Figure S2. Plot for median enantioselectivity for type of alkene substrates used with Ir, Rh, and Co metal catalysts in asymmetric hydrogenation of olefins. The identity of type of alkene substrates is shown in Figure 2a.

We also labelled each olefin type for all three metal catalysts based on median enantioselectivity (Figure S2). All olefin types in general show high median enantioselectivities with all three metal catalysts. The only prominent difference is observed in the case of enols where the Rh-catalyzed reactions provide a median enantioselectivity of 50. On the other hand, a high median enantioselectivity of 91.5 is observed with Ir catalysts.

3. Visualization of olefin chemical space

We visualized the chemical space covered by the olefins and ligands utilized in asymmetric hydrogenation catalyzed by all three metal catalysts. For this purpose, we used the UMAP (Uniform Manifold Approximation and Projection) plot. The UMAP plot for olefins is shown in Figures S3.

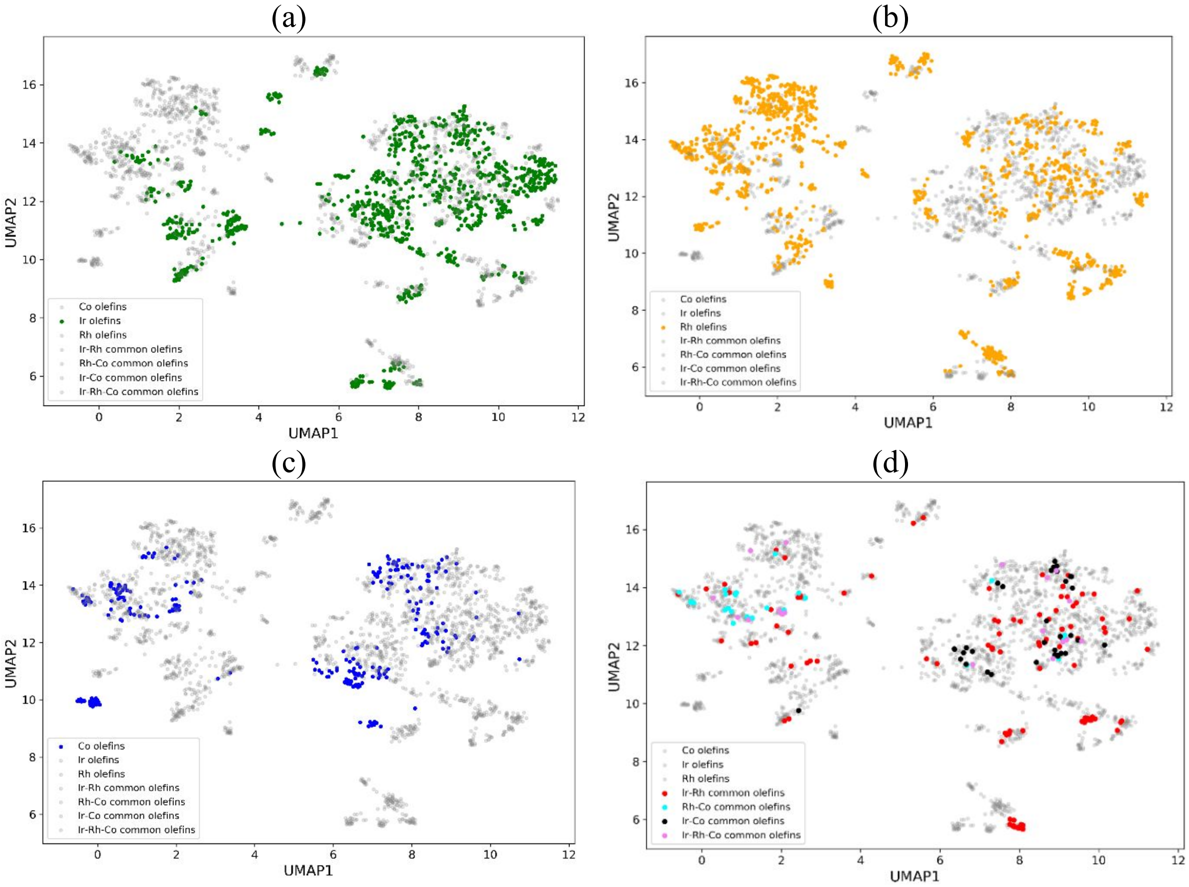

Figure S3. The UMAP plot of the chemical space of olefins unique to (a) iridium, (b) rhodium, (c) cobalt, and (d) common among metal catalysts as used in asymmetric hydrogenation.

We also visualized the chemical space covered by the olefins based on their average enantioselectivity values. For this purpose, we used the UMAP (Uniform Manifold Approximation and Projection) plot. The UMAP plot for olefins labelled by their enantioselectivity is shown in Figure S4. The chemical space of olefins corresponding to the three metal catalysts is shown in Figure 3. No significant trend is observed in Figure S4 based on the enantioselectivity values. Since the reaction data is skewed towards reactions with high enantioselectivity, same can be noted from Figure S4.

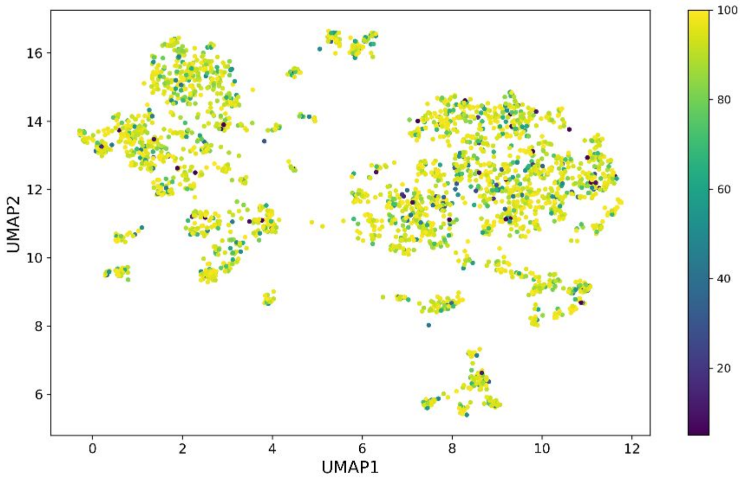

Figure S4. The UMAP plot of the chemical space of olefins labelled based on the average enantioselectivity in AHO reactions.

4. Diversity of ligands

The ten most-used phosphine-oxazoline ligands in Ir-catalyzed asymmetric hydrogenation of olefins is shown in Table S2.

Table S2. Top-10 Phosphine-oxazoline Ligands Utilized in Iridium-catalyzed Asymmetric Hydrogenation of Olefins. The Number of Reactions for a Given Ligand is Shown in Parentheses

Phosphine-oxazoline ligands		
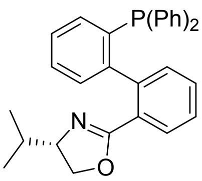	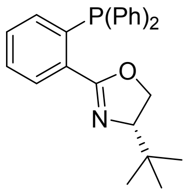	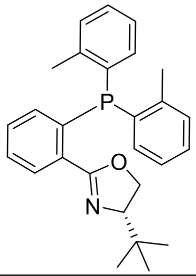
(91)	(90)	(77)
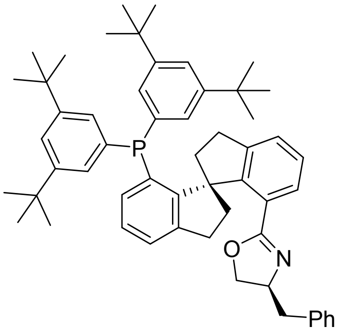	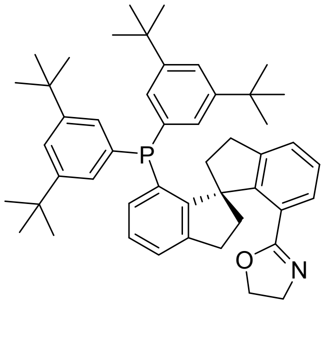	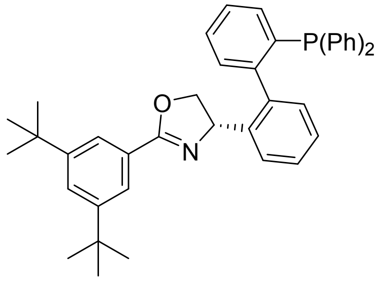
(76)	(58)	(42)
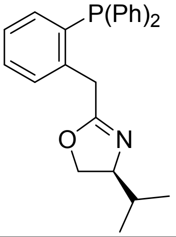	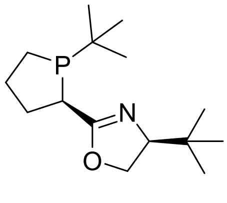	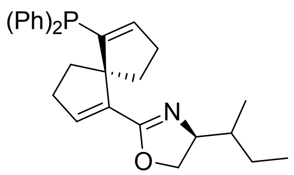
(36)	(34)	(31)
	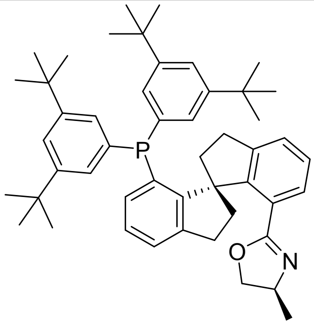	
	(30)	

The top-10 of the three most successful ligands in Rh-catalyzed AHO reactions, namely, bisphosphine, phosphoramidite, and ferrocenyl bisphosphine are shown in Table S3.

Table S3. Top-10 Bisphosphine, Phosphoramidite, and Ferrocenyl Bisphosphine Ligands Utilized in Rhodium-catalyzed Asymmetric Hydrogenation of Olefins. The Number of Reactions for a Given Ligand is Shown in Parentheses

Bisphosphines		
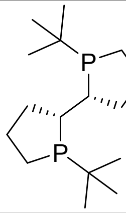	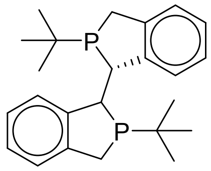	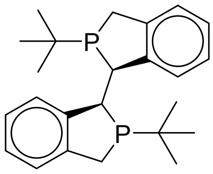
(190)	(123)	(118)
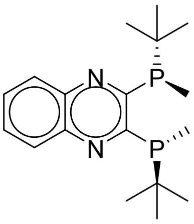	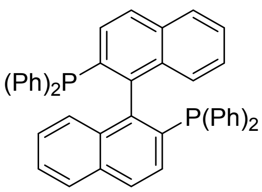	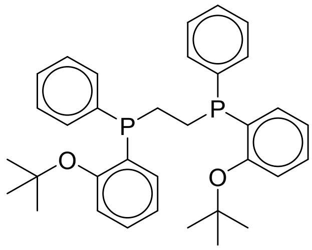
(74)	(71)	(53)
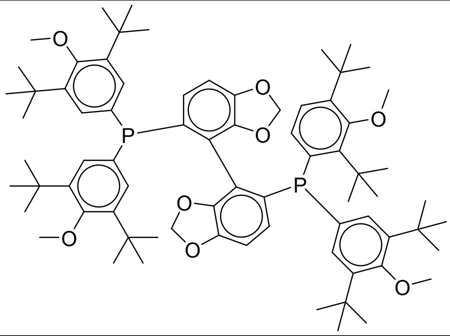	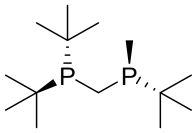	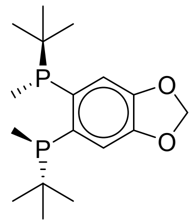
(52)	(48)	(42)
	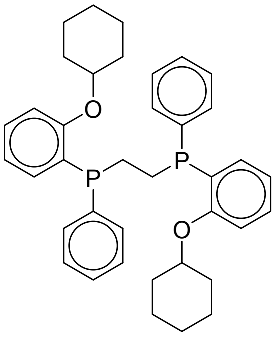	
	(40)	
Phosphoramidites		
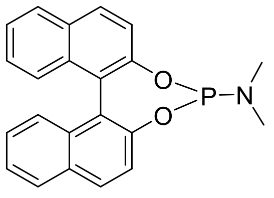	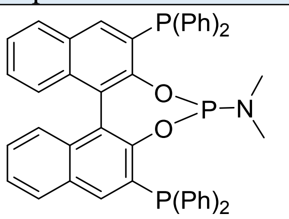	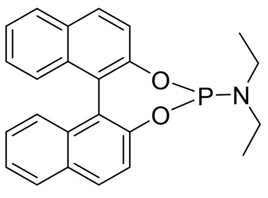
(267)	(82)	(51)

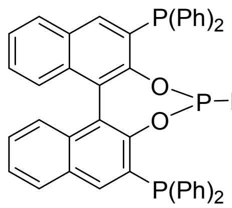	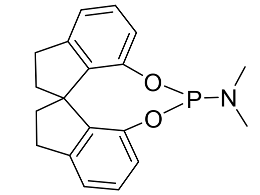	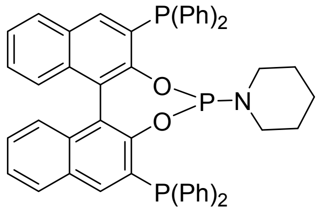
(45)	(45)	(44)
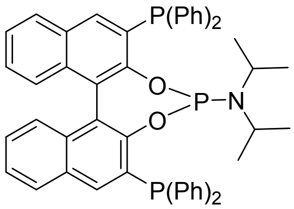	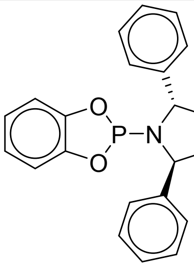	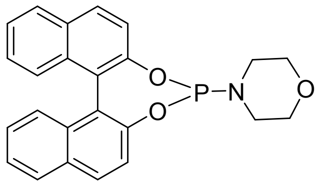
(44)	(42)	(41)
	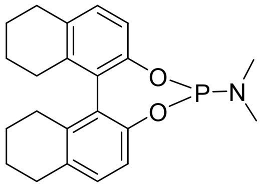	
	(41)	
Ferrocenyl bisphosphines		
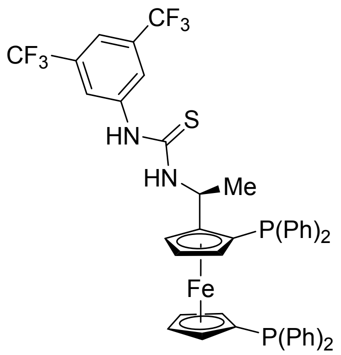	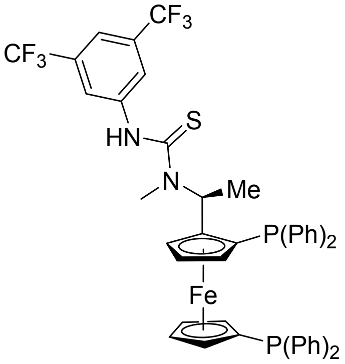	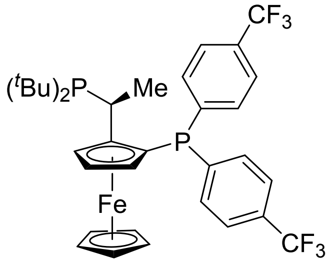
(545)	(74)	(54)
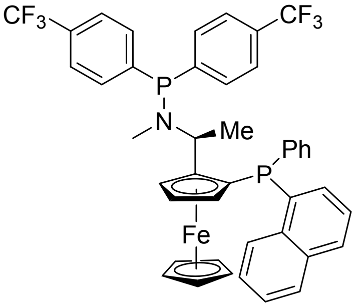	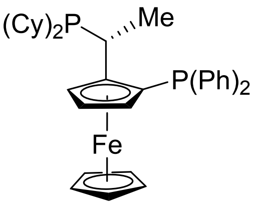	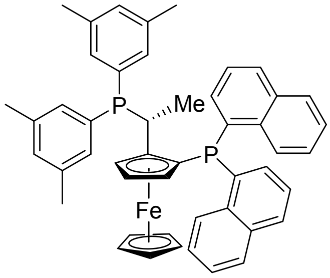
(41)	(40)	(32)
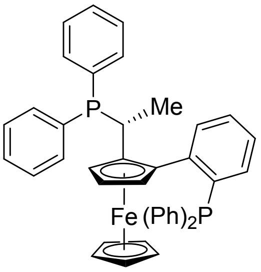	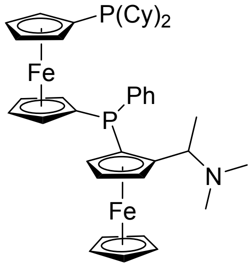	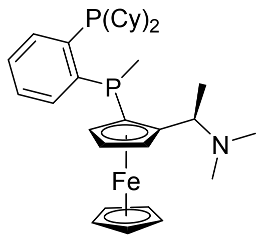

(26)	(24)	(23)
	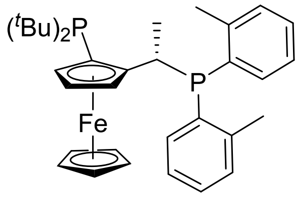	
	(19)	

The top-5 ligands with Co metal are shown in Table S4.

Table S4. Top-5 Ligands Utilized in Cobalt-catalyzed Asymmetric Hydrogenation of Olefins. The Number of Reactions for a Given Ligand is Shown in Parentheses

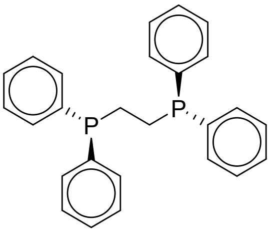	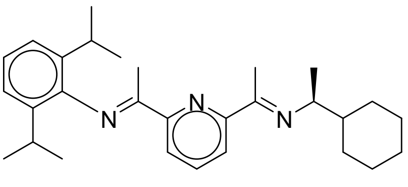	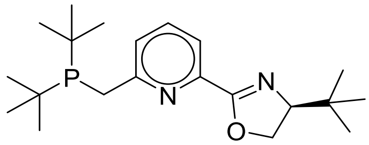
(212)	(38)	(37)
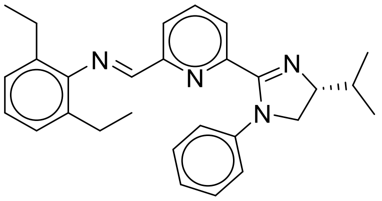	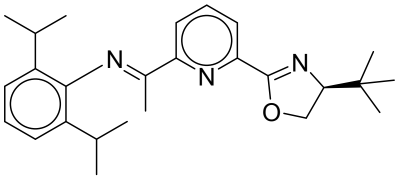	
(36)	(35)	

The number of unique ligands for each metal catalyst as well as the common ligands are shown in Table S5. The number of ligands common among different metal catalysts is limited. The type of ligands used with Ir and Rh catalysts that are common to Co includes bisphosphines. The most common ligand types between Ir and Rh are bisphosphines and phosphoramidites.

Table S5. The Number of Ligands Corresponding to Each Metal Catalyst

S. No.	Ligand type	Number of ligands
1.	Unique ligands with Ir	752
2.	Unique ligands with Rh	659
3.	Unique ligands with Co	43
4.	Common ligands with Ir and Rh	47
5.	Common ligands with Rh and Co	10
6.	Common ligands with Ir and Co	1
7.	Common ligands with Ir, Rh, and Co	5

5. Median enantioselectivity of different ligand types

We also labelled each ligand type for all three metal catalysts based on median enantioselectivity (Figure S5). There is no specific trend observed in the median enantioselectivity of P,N ligands (Figure S5(a)). Among the P,N-ligands, P-oxazolines in general have high median enantioselectivities with phosphine- and N-phosphine-oxazolines having a median enantioselectivity of 92. The ferrocene monophosphine and P, carbene ligands show significantly lower median enantioselectivities. The ferrocene bisphosphines and P,S ligands have median enantioselectivity lower than 80, while monophosphines/phosphinites are among the more successful ligands with median enantioselectivity of 95.

(a)

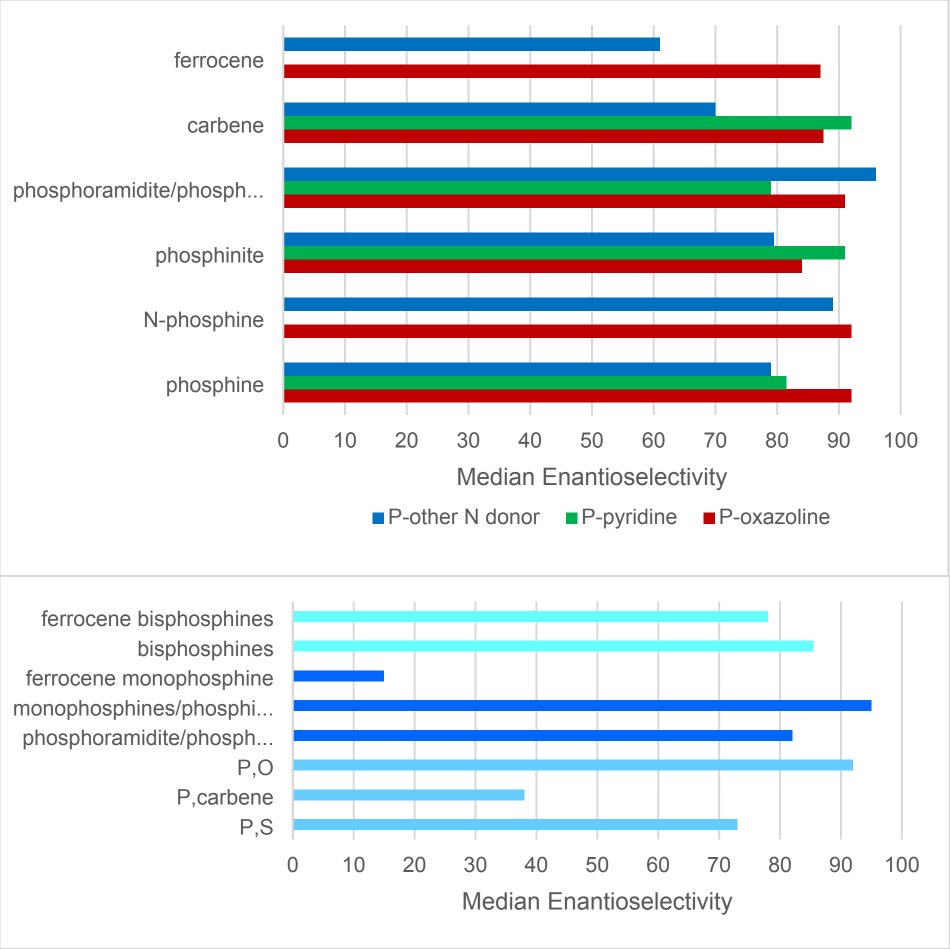

(b)

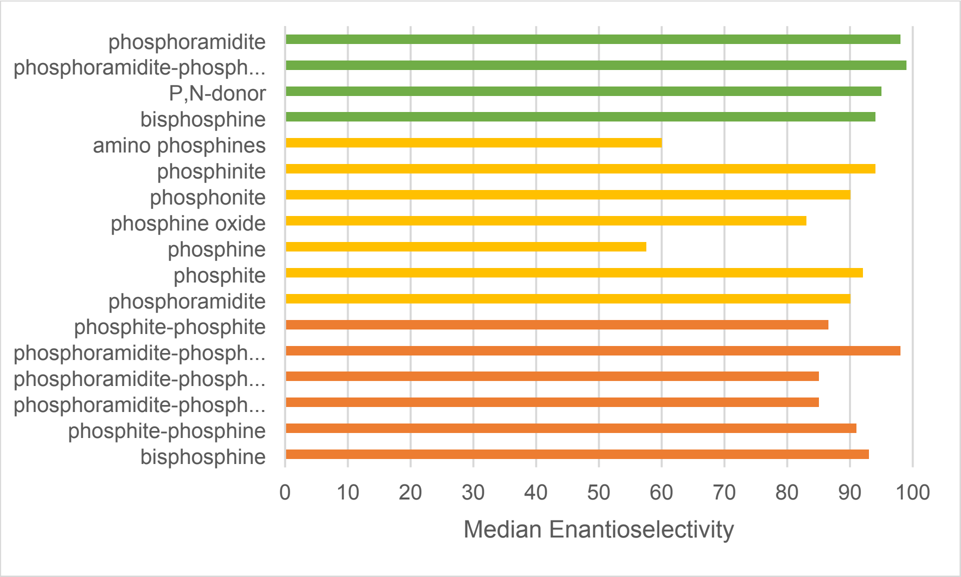

(c)

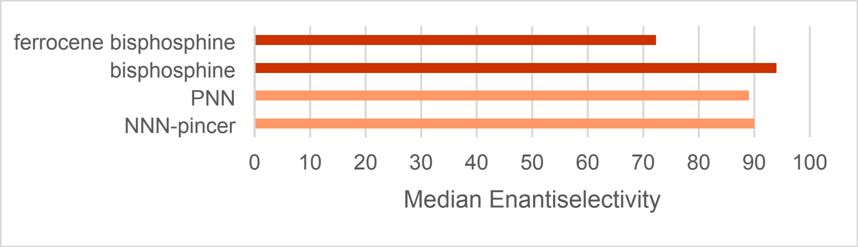

Figure S5. Plot for median enantioselectivity for type of ligands used with (a) Ir, (b) Rh, and (c) Co metal catalysts in asymmetric hydrogenation of olefins. The identity of type of ligands is shown in Figures 4b and 5.

In case of ligands used in Rh-catalyzed AHO reactions, the ferrocene-ligands have an overall high median enantioselectivity values (Figure S5(b)). Among mono-P ligands, phosphines and aminophosphines have median enantioselectivity of less than 60. All other mono-P ligands have median enantioselectivity over 80, with phosphinites having the highest enantioselectivity of 94. For P,P-ligands, all different ligand have very high median enantioselectivities with the maximum of 98 for phosphoramidite-phosphine. The ligand types used in Co-catalyzed AHO reactions have also median enantioselectivities greater than 80, with ferrocene bisphosphines as exception which have a median enantioselectivity of 72.3 (Figure S5(c)).

6. Visualization of ligand chemical space

We visualized the chemical space covered by the ligands utilized in asymmetric hydrogenation catalyzed by all three metal catalysts. For this purpose, we used the UMAP (Uniform Manifold Approximation and Projection) plot. The UMAP plot for olefins is shown in Figures S6.

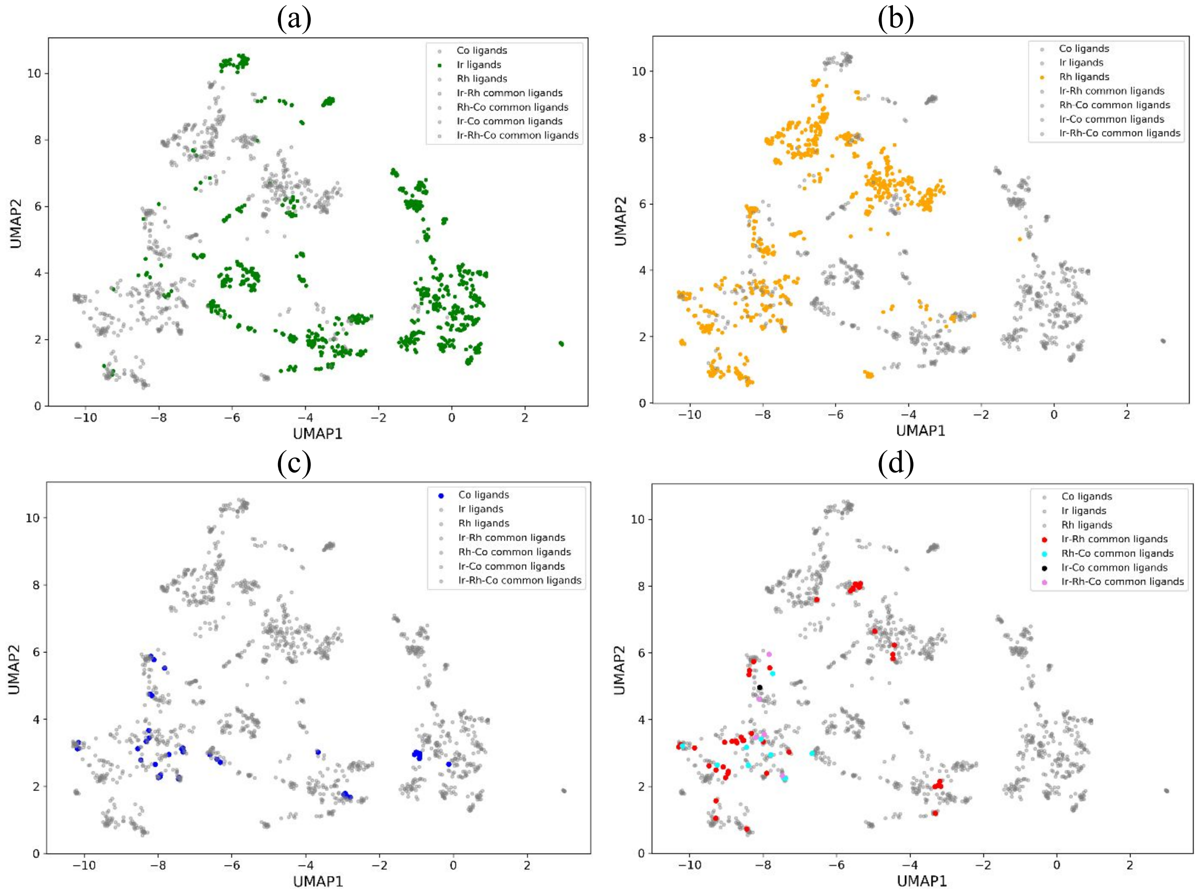

Figure S6. The UMAP plot of the chemical space of ligands unique to (a) iridium, (b) rhodium, (c) cobalt, and (d) common among metal catalysts as used in asymmetric hydrogenation of olefins.

We also visualized the chemical space covered by the ligands based on their average enantioselectivity values. For this purpose, we used the UMAP (Uniform Manifold Approximation and Projection) plot. The UMAP plot for ligands is shown in Figure S7. The chemical space of ligands corresponding to the three metal catalysts is shown in Figure 6. No significant trend is observed in Figure S7 based on the enantioselectivity values. Since the reaction data is skewed towards reactions with high enantioselectivity, same can be noted from

Figure S7. But, more variation in the enantioselectivity is noted with the ligand space as compared to the olefin chemical space.

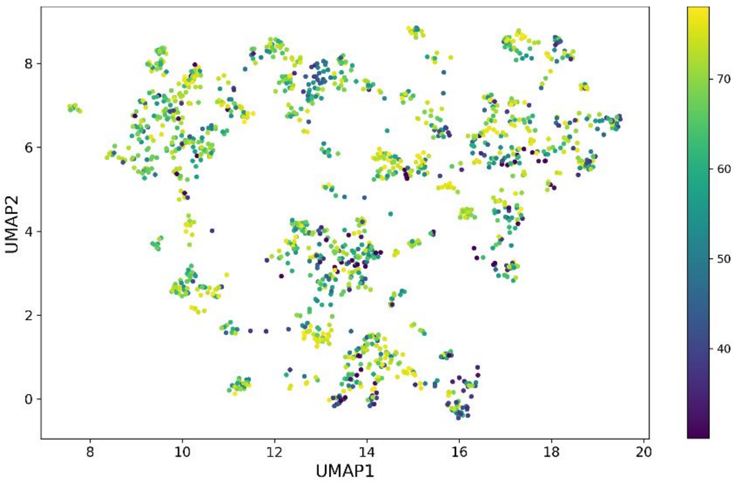

Figure S7. The UMAP plot of the chemical space of ligands labelled based on the average enantioselectivity in AHO reactions.

7. Visualization of olefin and ligand chemical space using t-SNE

We also visualized the chemical space covered by olefins and ligands used in AHO reaction using the t-distributed neighbor embedding (t-SNE) algorithm. It is a nonlinear dimensionality reduction technique suitable for visualizing high-dimensional data in two- or three-dimensions. The t-SNE plot for olefins is shown in Figure S8(a). The corresponding UMAP plot is presented in Figure 3. It can be noted from Figures S8(a) and 3 that the regions of chemical space occupied by various olefins is similar for both t-SNE and UMAP plots.

The t-SNE plot for ligands is shown in Figure S8(b). The corresponding UMAP plot is presented in Figure 6. Similar to olefins, the regions of chemical space occupied by various ligands is similar for both t-SNE and UMAP plots. A clear separation of ligands used in Ir- and Rh-catalyzed AHO reactions can be observed from t-SNE (Figure S8(b)) and UMAP (Figure 6) plots.

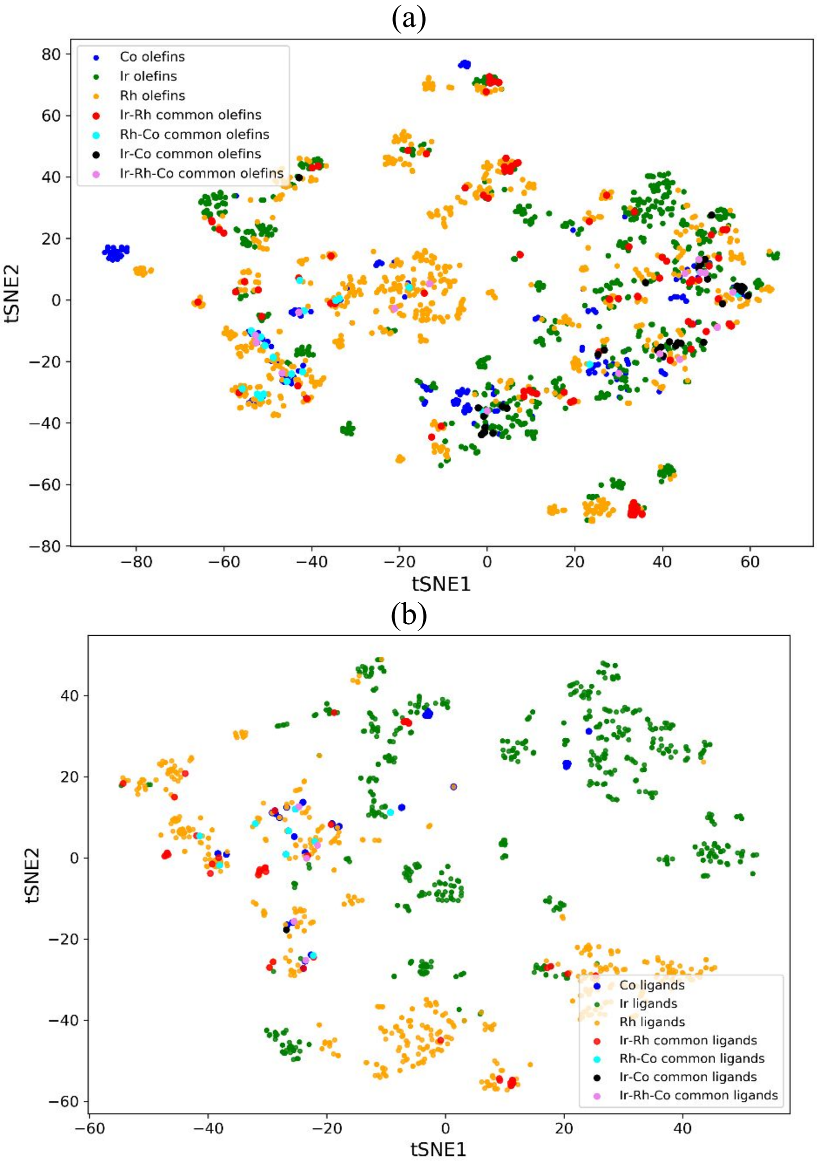

Figure S8. The t-SNE plot of the chemical space of (a) olefins and (b) ligands used in Ir-, Rh-, and Co-catalyzed asymmetric hydrogenation. The corresponding UMAP plots are shown in Figures 3 and 6 respectively.
